# Quality characterization of fermented rice wines: A multivariate study on physicochemical indices, biogenic amines, volatile profiles, and sensory evaluation

**DOI:** 10.1016/j.fochx.2026.104364

**Published:** 2026-08-24

**Authors:** HuaWei Yuan, XinYu Zhao, Hao Yuan, ZiYing Tian, Zhou Xu, TingNa Xie, Kai Lou

**Affiliations:** Faculty of Quality Management and Inspection & Quarantine / Solid-State Fermentation Resource Utilization Key Laboratory of Sichuan Province / Engineering Research Center of Agri-food Standardization and Inspection of the Education Department of Sichuan Province, Yibin University, Yibin, Sichuan, China

**Keywords:** Fermented rice wine, Physicochemical indices, Volatile flavor compounds, Sensory evaluation, Biogenic amines

## Abstract

This study comprehensively evaluated seven Chinese fermented rice wines by integrating physicochemical indices, free amino acids, organic acids, biogenic amines (BAs), volatile compounds, and sensory scores to identify key quality determinants. Results revealed substantial regional variations. Sample MJ5 (Guangxi) achieved the highest sensory acceptance (93.6 points), attributed to a harmonious balance of high alcohol content, umami-enhancing amino acids, and abundant floral/fruity esters. Conversely, MJ6 (Hubei) displayed comparable flavor complexity but carried the highest BA load, raising safety concerns, while MJ2 (Chongqing) exhibited the safest profile yet was sensorially plain. Notably, no single parameter dictated overall quality; instead, optimal rice wine requires synergistic integration of volatile and taste-active components alongside rigorous BA control. These findings provide a scientific foundation for establishing multi-parameter quality standards for fermented rice wines

## Highlights

• Seven commercial Chinese fermented rice wines were comprehensively characterized.
• Physicochemical indices, volatiles, biogenic amines and sensory scores were analyzed.
• Guangxi rice wine (MJ5) showed the highest sensory score due to balanced esters and umami amino acids.
• Hubei rice wine (MJ6) had complex flavor but contained the highest biogenic amines (132.54 mg/L).
• Optimal rice wine quality requires integration of aroma, taste components and controlled biogenic amines.

## 1 Introduction

Fermented rice wine, known as one of the oldest traditional alcoholic beverages in China, has been an integral part of Asian dietary culture for thousands of years. This distinctive beverage is produced through the fermentation of various cereals, primarily glutinous rice, using complex starter cultures such as *Qu* that contain diverse assemblages of microorganisms including molds, yeasts, and bacteria (Liu et al., 2022; Peng et al., 2025). The resulting product is characterized by its smooth mouthfeel, low alcohol content, and rich nutritional profile, which includes oligosaccharides, polypeptides, amino acids, vitamins, and minerals. These nutritional attributes, combined with its unique flavor profile, have contributed to the widespread popularity of fermented rice wine among consumers not only in China but also in other parts of the world (Koay et al., 2024; Wong et al., 2023). The sensory appeal of rice wine derives from the intricate balance of volatile and non-volatile compounds generated during the fermentation process, including esters, alcohols, organic acids, amino acids, and phenolic compounds, which collectively contribute to its characteristic aroma and taste (Chen et al., 2018; Yu et al., 2019).

Despite its long history and cultural significance, the Chinese rice wine industry faces considerable challenges related to product standardization and quality consistency. The market encompasses a diverse array of rice wine products manufactured using different raw materials, fermentation technologies, and production scales, ranging from traditional household brewing to modern industrial manufacturing (Lin et al., 2021; Yang, Xia, Wang, Hu, et al., 2017). This diversity, while contributing to the richness of rice wine varieties, has resulted in considerable variability in product quality and compositional characteristics. Studies have demonstrated that the concentrations of flavor compounds such as esters, amino acids, phenolic acids, and organic acids vary significantly depending on the raw materials and fermentation conditions employed (Shen et al., 2024; Zhao et al., 2020). For instance, Shen et al. (2024) reported that buckwheat-fermented rice wine exhibited the highest ester content among five different cereal-based wines, while glutinous rice wine showed the highest organic acid concentration. Such variability, although inherent to traditional fermented products, poses significant obstacles to the establishment of comprehensive quality standards and the consistent delivery of products that meet consumer expectations.

The quality of fermented rice wine is determined by a complex interplay of physicochemical parameters and flavor-active compounds. Key indicators such as total acid, total sugar, alcohol content, reducing sugar, and total soluble solids serve as fundamental quality markers that influence both the stability and sensory characteristics of the final product (Qian et al., 2023; Yang et al., 2021). During the fermentation process, dynamic changes occur in these parameters; for example, total sugar content typically decreases while alcohol and total acid contents increase as fermentation progresses (Liu et al., 2022; Zhao et al., 2023). Beyond these basic indices, amino acids and organic acids play particularly important roles in shaping the taste profile of rice wine. Amino acids contribute not only to nutritional value but also to taste characteristics, with bitter amino acids often comprising more than 50% of the total amino acid content in some rice wine varieties (Deng et al., 2025; Shen et al., 2024). Organic acids, derived from both raw materials and microbial metabolism during fermentation, influence the acidity, buffering capacity, and overall flavor balance of the final product (Yang, Li, et al., 2024; Huang et al., 2023). The complex interactions among these components ultimately determine the sensory quality and consumer acceptability of rice wine.

Recent advances in analytical methodologies have enabled more comprehensive characterization of rice wine composition and its relationship to sensory quality. Headspace solid-phase microextraction coupled with gas chromatography–mass spectrometry (HS-SPME-GC–MS) has been widely employed to identify volatile flavor compounds, while high-performance liquid chromatography (HPLC) facilitates the quantification of non-volatile components such as organic acids and amino acids (Liu et al., 2025; Yang, Xia, Wang, Hu, et al., 2017). Studies utilizing these techniques have revealed the presence of numerous flavor-active compounds in rice wine, including esters, alcohols, aldehydes, acids, and phenolic compounds (Cui et al., 2024; Yu et al., 2019). The application of odor activity values (OAV) has further enabled the identification of key aroma compounds that contribute most significantly to the overall flavor profile (Chen et al., 2013; Jiao et al., 2025). Deng et al. (2025) demonstrated that different fermentation microorganisms can substantially influence the volatile organic compound profile, with certain non-*Saccharomyces* yeasts enhancing the production of pleasant aroma compounds while reducing the content of bitter amino acids. These methodological advances provide powerful tools for establishing correlations between chemical composition and sensory attributes, thereby supporting quality improvement and standardization efforts.

The relationship between physicochemical parameters and sensory quality has been the subject of increasing research attention, yet substantial knowledge gaps remain. While individual studies have examined specific aspects of rice wine composition, integrated investigations that simultaneously analyze multiple physicochemical indices, flavor components, and sensory attributes are relatively scarce (Yu et al., 2022; Zhang et al., 2024). Furthermore, the lack of comprehensive quality standards for fermented rice wine in China has resulted in products with inconsistent quality characteristics, potentially undermining consumer confidence and limiting market development (Peng et al., 2025; Yuan et al., 2025). Sensory evaluation, which integrates olfactory, gustatory, and tactile perceptions, provides a holistic assessment of rice wine quality that complements instrumental analysis (Koay et al., 2024; Wong et al., 2023). However, systematic studies correlating comprehensive physicochemical profiles with sensory evaluation results across different commercial rice wine products are needed to establish meaningful quality benchmarks.

Although numerous studies have separately investigated the physicochemical properties, volatile profiles, or sensory characteristics of fermented rice wines, a comprehensive analysis that simultaneously examines all these parameters—together with free amino acids, organic acids, and biogenic amines—within a single set of commercial samples remains scarce. This study addresses this gap by adopting a holistic analytical framework that integrates multiple quality indicators and directly correlates them with sensory evaluation data. Our work offers several novel contributions: (i) it provides a systematic quality comparison of seven geographically distinct commercial rice wine products, reflecting real-world variability that laboratory-scale fermentations cannot capture; (ii) it quantifies the critical trade-off between flavor complexity (driven by esters and higher alcohols) and food safety (reflected by biogenic amine accumulation), revealing that high sensory scores do not necessarily accompany low safety risks; (iii) it employs multivariate statistical approaches to identify the specific compounds that most strongly influence consumer acceptance, thereby offering actionable targets for quality control; and (iv) it establishes a scientific rationale for moving beyond single-parameter quality assessment toward a multi-indicator standardization system. Accordingly, the specific objectives of this study were to comprehensively characterize the physicochemical, amino acid, organic acid, biogenic amine, and volatile profiles of seven commercial rice wines, correlate these chemical parameters with sensory evaluation scores to identify key quality drivers, and provide a scientific basis for establishing multi-parameter quality standards that balance flavor complexity with safety considerations, ultimately supporting product consistency and sustainable development of the rice wine industry.

## 2 Materials and methods

### 2.1 Collection of fermented rice wine samples

Fermented rice wine samples were collected from seven rice wine manufacturers located in Anyuan County, Jiangxi Province; Chongqing City; Dazhou City, Sichuan Province; Jinping County, Guizhou Province; Laibin City, Guangxi Zhuang Autonomous Region; Xiaogan City, Hubei Province; and Yibin City, Sichuan Province, China. The samples were sequentially labeled MJ1, MJ2, MJ3, MJ4, MJ5, MJ6, and MJ7. Despite the general consistency in the basic production workflow—including steaming, cooling, starter mixing, saccharification and fermentation, filtration, and clarification, without any distillation or blending—across all manufacturers, notable differences in raw materials, processing parameters, and storage conditions cannot be excluded. For instance, variations in glutinous rice cultivars, the microbial and enzymatic composition of *Qu* starters, fermentation temperature and duration, as well as post-fermentation aging and storage practices, may substantially influence the final physicochemical and sensory profiles. However, these specific parameters were not disclosed by the manufacturers owing to proprietary protocols, which constitutes a limitation of this study and underscores the need for future research that systematically monitors these variables to better elucidate their individual contributions to product quality and safety. All samples were collected in March 2025 and stored at −18 °C for up to 10 days prior to analysis.

### 2.2 Pretreatment of fermented rice wine

After opening, samples were centrifuged (Centrifuge: TGL-20 M, Luxiangyi, Shanghai, China; 5000 ×g, 10 min, 4 °C). The supernatant was transferred into pre-sterilized 50-mL amber glass bottles, sealed with PTFE/silicone septa, and stored at 4 °C in darkness. All analyses were completed within 7 days post-opening to prevent oxidation or microbial spoilage. For assays requiring particle-free solutions, samples were filtered through 0.22 μm nylon membrane filters (Jinteng, Tianjin, China) or diluted 1:5 to 1:20 (*v*/v) with ultrapure water (18.2 MΩ·cm, Milli-Q®).

### 2.3 Measurement of physicochemical indices

The ethanol content was measured using a fully automatic density-refractometer (RM40, Mettler Toledo) after distillation. Total acid (titratable acidity) was determined by titration with 0.1 M NaOH to pH 8.2 (pH meter: FiveEasy™ FE28, Mettler Toledo), expressed as lactic acid equivalents (g/L), following the method of Chen et al. (2020). Volatile acids were quantified by steam distillation followed by titration (GB/T 15038—2006, General Administration of Quality Supervision, Inspection and Quarantine of China, & Standardization Administration of China, 2006). Reducing sugar was analyzed by the DNS colorimetric method (He et al., 2022) using a UV–Vis spectrophotometer (Meixi UV-1500, Shanghai, China) at 540 nm against a glucose standard curve (0–1.0 mg/mL, R^2^ = 0.999). Amino acid nitrogen (AAN) was determined by formol titration (GB 5009.235—2016; National Health Commission of China and Standardization Administration of China, 2016a) with 0.05 M NaOH.

### 2.4 Determination of amino acids in fermented rice wine

Free amino acids were quantified using an automatic amino acid analyzer (Sykam S433, Munich, Germany) following the method of Yan et al. (2021) with minor modifications. Samples were deproteinized by mixing 1 mL of supernatant with 1 mL of 5% sulfosalicylic acid, vortexed for 30 s, incubated at 4 °C for 1 h, and then centrifuged at 10,000 ×*g* for 15 min at 4 °C. The clear supernatant was filtered through 0.22-μm filter membraneChromatographic separation was performed on LCA K07/Li cation-exchange resin column (150 × 4.6 mm, 7 μm) using gradient elution with lithium citrate buffers (pH 2.2 and 4.3) at a flow rate of 0.4 mL/min, with the column oven at 57 °C and the reaction coil at 135 °C. Post-column ninhydrin derivatization enabled detection at 570 nm (primary amines) and 440 nm (proline). Quantification was carried out using external standard curves (0.1–50 μmol/L) prepared from 17 amino acid standards (Sigma-Aldrich, purity ≥ 99%), with correlation coefficients (R^2^) exceeding 0.995 for all analytes.

### 2.5 Determination of organic acids in fermented rice

The organic acids—including oxalic, acetic, lactic, malic, succinic, citric, and tartaric acids—were qualitatively and quantitatively determined following the method of Yang et al. (2018) with appropriate modifications, using Shimadzu LC-20 A HPLC equipped with Inertsil ODS-SP C18 column (250 × 4.6 mm, 5 μm). The mobile phase was 5 mM H₂SO₄ at an isocratic flow rate of 0.6 mL/min, with the column temperature maintained at 60 °C and UV detection at 210 nm. Prior to injection, samples were filtered through 0.22 μm membranes and diluted 10-fold. Quantification was performed using external standards (Sigma-Aldrich) over a linear range of 1–200 mg/L, with all calibration curves yielding R^2^ > 0.99.

### 2.6 Determination of biogenic amines in fermented rice wine

The determination of biogenic amines was carried out according to GB 5009.208—2016 (National Health Commission of China and Standardization Administration of China, 2016b), using dansyl chloride derivatization. The derivatized samples were analyzed by HPLC (Shimadzu LC-20 A) equipped with a ZORBAX Eclipse Plus C18 column (250 × 4.6 mm, 5 μm) and a gradient elution program with mobile phase A (0.1 M ammonium acetate) and B (acetonitrile). Fluorescence detection (FLD) was performed at excitation and emission wavelengths of 254 nm. The method exhibited satisfactory recovery rates of 92–105% in spiked samples.

### 2.7 Analysis of volatile flavor compounds

Volatile compounds in the rice wine samples were analyzed by headspace solid-phase microextraction coupled with gas chromatography–mass spectrometry (HS-SPME-GC–MS). Briefly, each sample (3.0 mL) was mixed with 1.2 g NaCl in a 15-mL headspace bottle, and 10 μL of 2-octanol (0.0716 mg/mL in methanol) was added as the internal standard. The vial was pre-equilibrated in a water bath at 60 °C for 15 min, and volatiles were then extracted at the same temperature for 45 min using a 50/30 μm DVB/CAR/PDMS fiber (Supelco, Bellefonte, PA, USA). The extracted compounds were desorbed and analyzed on a Shimadzu QP2020 GC–MS system equipped with an HP-INNOWax column (60 m × 0.25 mm i.d., 0.25 μm film thickness, J&W Scientific). Ultra-high-purity helium was used as the carrier gas at a constant flow of 1.0 mL/min. The oven temperature was programmed as follows: held at 40 °C for 5 min, raised to 100 °C at 5 °C/min, then increased to 230 °C at 6 °C/min and held for 10 min. The transfer line and ion source temperatures were set to 250 °C and 230 °C, respectively. Compound identification was performed by matching mass spectra against the NIST-14.0 library (match >85%), and semi-quantification was achieved using the internal standard method based on 2-octanol, following the procedure described by Xiao et al. (2021).

### 2.8 Sensory properties analysis

Sensory evaluation of the rice wine samples was conducted by a trained panel consisting of twelve judges (six females and six males, aged 20 to 50 years), including rice wine critics and professional rice wine technicians, following the guidelines of ISO 11035 (International Organization for Standardization, 1994) and similar to the approach described by Wang et al. (2013). Fourteen sensory descriptors were selected to characterize the samples, covering aroma (alcoholic, fruity, honey, floral, cereal), taste (sweetness, acidity, astringency, umami, bitterness), mouthfeel (continuation, fullness), and appearance (yellowness, turbidity). Each descriptor was rated on a nine-point intensity scale (0 = none, 1–2 = very weak, 3–4 = ordinary, 5–6 = moderate, 7–8 = intense; 9 = very intense). A 100-point scoring system was also applied, allocating 60 points to taste, 20 points to aroma, 10 points to color, and 10 points to full-body. A total score of 100 indicated outstanding quality.

In accordance with earlier reports (Yuan et al., 2022), the sensory tests were performed in a dedicated testing room maintained at 20 °C, equipped with uniform lighting, and free from noise or other distracting stimuli. Blind assessments were performed using randomly coded samples. The panelists vigorously swirled each cup and inhaled the headspace vapor to evaluate the aroma. Rice wine samples (30 mL) were poured into identical cups, served at 20 °C, and presented to the judges with only the sample codes visible. Between samples, panelists rinsed their mouths with water. A reference card listing all sensory attributes along with their definitions and the nine-point intensity scale (0–9) was provided to assist the tasting. The judges were asked to rate each descriptor for every sample.

### 2.9 Statistical analysis

All analyses were performed in triplicate. Results were examined by one-way analysis of variance (ANOVA) employing SPSS (version 22.0, IBM Corp., Armonk, NY, USA). Using the Duncan test at *p* < 0.05, significant variations across the data were found. The findings were presented as mean ± standard deviation (SD).

## 3 Results and discussion

### 3.1 Physicochemical properties and sensory quality of fermented rice wine

Before evaluating the individual samples, it is important to define what constitutes ‘good quality’ in the context of this study. Based on our integrated analytical framework and sensory validation, we considered a rice wine to be of good quality when it exhibited: (1) a high overall sensory score (> 90 points), reflecting consumer acceptance; (2) a chemically complex and balanced profile—characterized by abundant esters and higher alcohols, moderate-to-high umami amino acids, and a well-structured organic acid composition; (3) biogenic amine levels within acceptable safety limits (provisionally <100 mg/L total BAs), and (4) harmonized integration of these attributes rather than excellence in any single parameter. This multi-dimensional definition guided our comparative assessment of the seven commercial samples.

The fundamental physicochemical parameters and sensory scores of the seven rice wine samples (MJ1–MJ7) are presented in Table 1(Supplementary Table S1 and S2). These indices directly reflect the metabolic activities of the microbial community and significantly influence the final product's quality and consumer acceptance (Peng et al., 2025; Yang, Xia, et al., 2024).

**Table 1:** The physicochemical parameters and sensory scores of the seven rice wine samples.

	MJ1	MJ2	MJ3	MJ4	MJ5	MJ6	MJ7
Total Acidity (g/L)	3.23 ± 0.16^c^	1.90 ± 0.09^e^	1.99 ± 0.01^e^	2.74 ± 0.09^d^	5.53 ± 0.15^a^	5.02 ± 0.23^b^	2.51 ± 0.08^d^
Volatile acid (g/L)	0.18 ± 0.01^f^	0.23 ± 0.01^e^	0.24 ± 0.01^e^	0.50 ± 0.01^b^	0.67 ± 0.02^a^	0.43 ± 0.01^c^	0.32 ± 0.02^d^
Total sugar (g/L)	96.84 ± 0.13^g^	171.07 ± 0.91^c^	159.19 ± 0.32^d^	188.89 ± 0.29^b^	137.29 ± 0.19^e^	209.75 ± 0.47^a^	127.80 ± 0.83^f^
Reducing Sugar(g/L)	85.28 ± 0.18^e^	160.78 ± 0.61^a^	112.85 ± 0.62^b^	108.62 ± 0.56^c^	71.29 ± 0.64^f^	109.04 ± 1.01^c^	87.19 ± 0.89^d^
Amino acid nitrogen (mg/100 mL)	12.13 ± 0.20^e^	20.80 ± 0.59^c^	24.53 ± 0.74^b^	20.59 ± 0.43^c^	27.07 ± 0.40^a^	14.72 ± 0.02^d^	11.49 ± 0.39^e^
Alcohol Content (% vol)	6.81 ± 0.02^b^	4.45 ± 0.01^e^	4.43 ± 0.02^e^	5.42 ± 0.03^d^	8.35 ± 0.03^a^	5.43 ± 0.01^d^	6.14 ± 0.03^c^
Sensory score (points)	83.4 ± 2.2^d^	88.8 ± 2.4^c^	81.4 ± 2.6^e^	80.3 ± 2.4^e^	93.6 ± 1.8^a^	91.4 ± 2.5^b^	88.9 ± 1.4^c^

Note: values with distinct superscript letters within the same row were statistically significant (*p* < 0.05).All findings are shown as the mean ± standard deviation (*n* = 3).

The alcohol content, a primary indicator of fermentation efficiency, varied significantly (*p* < 0.05) among the samples, ranging from 4.43%vol (MJ3) to 8.35%vol (MJ5). MJ5 exhibited the highest alcohol content, which was consistent with its highest total acidity (5.53 g/L) and volatile acid (0.67 g/L) levels. This suggests a more vigorous fermentation process, potentially driven by a more robust yeast population, as *Saccharomyces cerevisiae* is known to be a primary producer of ethanol and organic acids during rice wine fermentation (Tang & Peng, 2024; Yang, Xia, Wang, Yu, & Ai, 2017). The high sensory score of MJ5 (93.6 points) corroborates that a balanced increase in these parameters contributes positively to the overall flavor profile. Conversely, MJ2 and MJ3 showed the lowest alcohol content (∼4.4%vol) and total acidity (∼1.9 g/L), which correlated with their lower sensory scores, indicating that insufficient fermentation leads to a flat and less desirable taste (Koay et al., 2024).

Total sugar and reducing sugar contents displayed a complex pattern. MJ6 had the highest total sugar (209.75 g/L), while MJ1 had the lowest (96.84 g/L). Interestingly, MJ1 also had the highest reducing sugar (85.28 g/L) as a proportion of its total sugar, indicating a more complete saccharification. The high sugar content in MJ6 did not translate into the highest alcohol, suggesting that its saccharification power may have exceeded the yeast's fermentation capacity or that the microbial community favored sugar accumulation over alcohol production. This observation aligns with studies showing that the balance between *Rhizopus* and *Saccharomyces* species dictates the final sugar-to-alcohol ratio (He et al., 2025; Zhao et al., 2023). The amino acid nitrogen (AAN) content, crucial for flavor and yeast nutrition, was highest in MJ5 (27.07 mg/100 mL) and lowest in MJ7 (11.49 mg/100 mL). High AAN contributes to the umami taste and serves as a precursor for higher alcohols, which explains the robust and complex flavor of MJ5 (Deng et al., 2025; Shi et al., 2022). The strong positive correlation between AAN, alcohol content, and sensory score across the samples underscores the importance of nitrogen metabolism for overall rice wine quality (Qian et al., 2023).

**Fig. 1:**
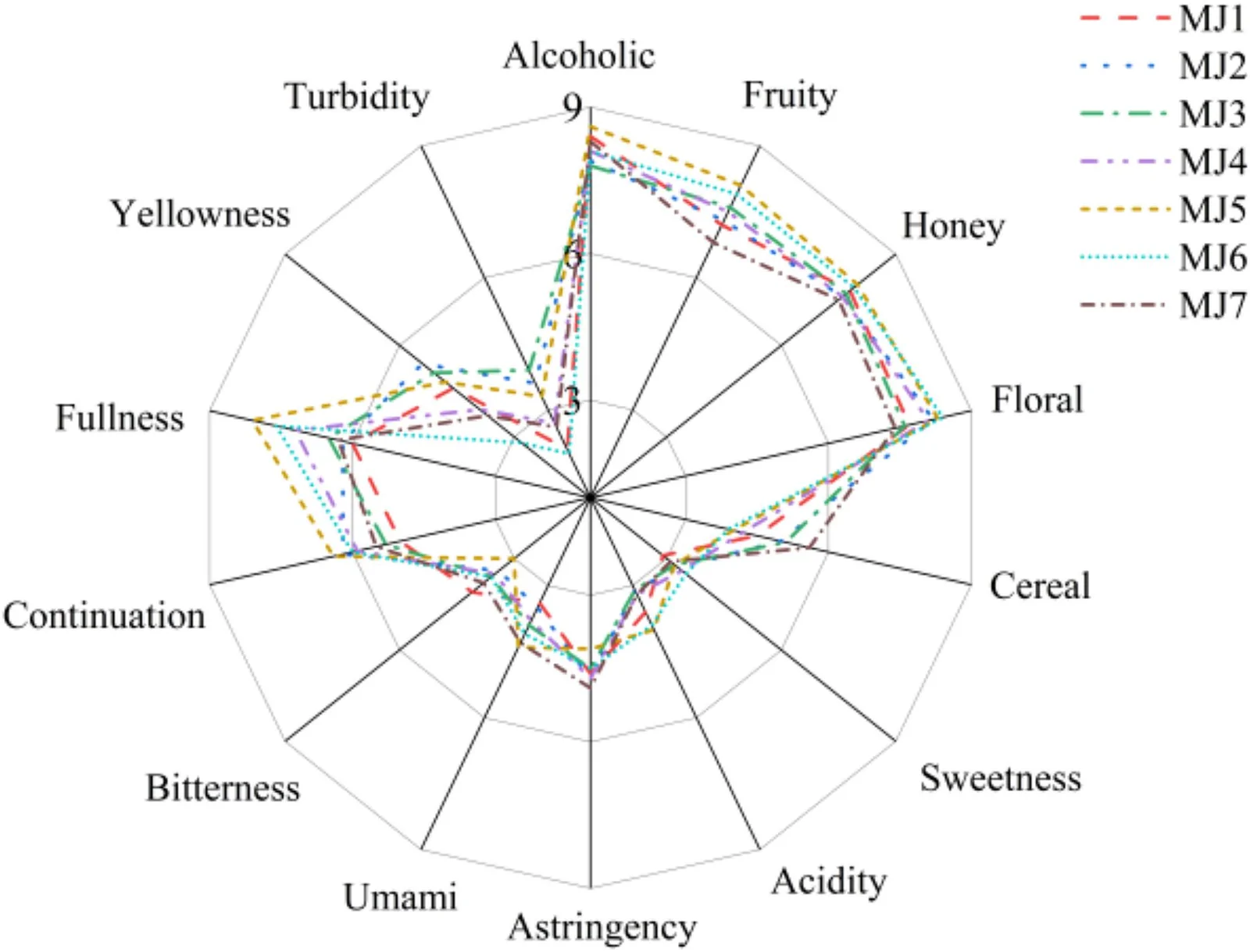
Radar chart of sensory evaluation scores for samples across 14 attributes.

The sensory evaluation, depicted by the radar chart, revealed a distinct shared flavor framework across all seven samples (MJ1–MJ7), characterized by high intensities in Alcoholic, Fruity, Floral, and Honey attributes (predominantly scoring above 6 on a 9-point scale) and uniformly low scores for Bitterness, Umami, Astringency, and Cereal (consistently hovering around 3), indicating a clean and aromatically driven profile (Fig. 1); however, the polygonal areas exhibited substantial inter-sample variation, with MJ5 demonstrating the most robust overall intensity—approaching the maximum score of 9 across multiple dimensions including Alcoholic, Fruity, Honey, Fullness, and Continuation—closely followed by MJ3, whereas MJ7 presented the weakest performance with the smallest radar envelope, particularly in the core aromatic attributes (Fig. 1).

Furthermore, MJ1 displayed a polarized profile characterized by high aromatic notes but notably lower Fullness and Yellowness, while MJ6 was distinctly differentiated by the highest Turbidity score and above-average Fullness, with MJ2 and MJ4 maintaining intermediate profiles (Fig. 1). Collectively, these findings establish MJ5 and MJ3 as the samples with the most comprehensive and intense sensory qualities, whereas MJ7 exhibits the lowest overall sensory strength, and the distinct features of MJ6 and MJ1 serve as key distinguishing factors within the analyzed group.

### 3.2 Free amino acid and organic acid profiles

Free amino acids (FAAs) are critical for both the nutritional value and the taste of rice wine, contributing to sweetness, bitterness, and umami (Shen et al., 2024). The total FAA content varied dramatically, with MJ7 showing the highest concentration (95.23 mg/100 mL), followed by MJ5 (88.53 mg/100 mL), while MJ1 had the lowest (39.44 mg/100 mL) (Table 2, Supplementary Table S3).

**Table 2:** Amino acid contents of different fermented rice wines (mg/100 mL).

	MJ1	MJ2	MJ3	MJ4	MJ5	MJ6	MJ7
Asp	3.81 ± 0.02^e^	6.41 ± 0.02^d^	6.45 ± 0.16^d^	6.35 ± 0.07^d^	8.98 ± 0.09^b^	6.82 ± 0.03^c^	9.15 ± 0.14^a^
Thr	1.62 ± 0.03^e^	2.40 ± 0.05^d^	2.35 ± 0.01^d^	2.43 ± 0.03^c^	3.48 ± 0.01^a^	2.70 ± 0.02^b^	3.54 ± 0.06^a^
Ser	2.18 ± 0.03^f^	3.37 ± 0.02^d^	3.24 ± 0.03^e^	3.36 ± 0.02^d^	4.87 ± 0.02^b^	3.81 ± 0.03^c^	5.09 ± 0.05^a^
Glu	8.25 ± 0.04^e^	12.48 ± 0.05^d^	12.49 ± 0.03^d^	12.40 ± 0.09^d^	19.08 ± 0.05^b^	14.06 ± 0.14^c^	19.40 ± 0.04^a^
Gly	2.43 ± 0.03^f^	3.16 ± 0.01^e^	3.16 ± 0.01^e^	3.28 ± 0.03^d^	5.05 ± 0.01^b^	3.64 ± 0.03^c^	7.32 ± 0.11^a^
Ala	2.39 ± 0.09^e^	3.64 ± 0.02^cd^	3.60 ± 0.02^d^	3.83 ± 0.02^c^	5.84 ± 0.03^b^	3.75 ± 0.04^cd^	6.56 ± 0.26^a^
Cys	0.57 ± 0.02^d^	0.56 ± 0.01^d^	0.51 ± 0.01^f^	0.53 ± 0.01^e^	1.15 ± 0.02^b^	0.76 ± 0.02^c^	1.22 ± 0.03^a^
Val	1.95 ± 0.04^e^	3.53 ± 0.05^d^	3.73 ± 0.03^c^	3.60 ± 0.02^d^	4.40 ± 0.06^b^	3.48 ± 0.16^d^	4.76 ± 0.05^a^
Met	0.15 ± 0.01^e^	0.23 ± 0.01^d^	0.23 ± 0.01^d^	0.23 ± 0.01^d^	0.40 ± 0.02^b^	0.30 ± 0.01^c^	0.75 ± 0.04^a^
Ile	1.40 ± 0.03^e^	2.56 ± 0.03^c^	2.72 ± 0.06^b^	2.65 ± 0.03^b^	3.04 ± 0.02^a^	2.36 ± 0.11^d^	3.11 ± 0.07^a^
Leu	2.33 ± 0.03^f^	4.44 ± 0.03^e^	4.51 ± 0.02^d^	4.65 ± 0.04^c^	5.05 ± 0.04^b^	4.41 ± 0.02^e^	5.13 ± 0.04^a^
Tyr	1.14 ± 0.02^f^	1.55 ± 0.01^c^	1.55 ± 0.02^c^	1.55 ± 0.03^c^	1.55 ± 0.04^c^	1.55 ± 0.05^c^	1.55 ± 0.06^c^
Phe	1.69 ± 0.02^e^	3.28 ± 0.02^d^	3.25 ± 0.04^d^	3.27 ± 0.03^d^	3.77 ± 0.03^b^	3.50 ± 0.08^c^	4.03 ± 0.07^a^
Lys	1.83 ± 0.03^f^	2.74 ± 0.05^e^	2.83 ± 0.08^d^	3.00 ± 0.02^c^	3.69 ± 0.05^b^	2.96 ± 0.06^c^	3.89 ± 0.02^a^
His	1.25 ± 0.02^e^	1.63 ± 0.02^d^	1.74 ± 0.06^c^	1.73 ± 0.04^c^	3.38 ± 0.01^a^	2.07 ± 0.03^b^	3.42 ± 0.05^a^
Arg	3.92 ± 0.08^f^	6.60 ± 0.03^e^	6.77 ± 0.04^d^	6.65 ± 0.06^e^	9.07 ± 0.02^b^	7.30 ± 0.07^c^	9.71 ± 0.03^a^
Pro	2.53 ± 0.09^e^	3.08 ± 0.02^d^	3.02 ± 0.07^d^	3.07 ± 0.02^d^	5.05 ± 0.06^b^	3.62 ± 0.02^c^	5.83 ± 0.02^a^
Total	39.44 ± 0.14^e^	61.64 ± 0.33^d^	62.15 ± 0.44^d^	62.42 ± 0.51^d^	88.53 ± 0.41^b^	66.81 ± 0.73^c^	95.23 ± 0.53^a^

Note: values with distinct superscript letters within the same row were statistically significant (*p* < 0.05).All findings are shown as the mean ± standard deviation (*n* = 3).

The high total FAA in MJ7 and MJ5 was primarily driven by elevated levels of umami-contributing amino acids like glutamic acid (Glu, 19.40 and 19.08 mg/100 mL, respectively) and aspartic acid (Asp, 9.15 and 8.98 mg/100 mL, respectively). These findings are significant because Glu and Asp are known to enhance the pleasant, full-bodied mouthfeel of fermented products (Cen et al., 2023; Shi et al., 2022). The presence of bitter amino acids, such as Val, Ile, and Leu, was also higher in MJ5 and MJ7. However, their high sensory scores suggest that these bitter notes were well-balanced by the high sugar and umami components, creating a complex and desirable flavor profile, a phenomenon also observed in other traditional rice wines (He et al., 2025; Qian et al., 2023). The lowest FAA content in MJ1 correlates with its lower sensory score, confirming that adequate proteolysis is essential for flavor development (Liu et al., 2022).

The organic acid composition, which influences the taste and microbial stability of the wine, also showed significant regional variation (Table 3, Supplementary Table S4). MJ5 and MJ6 were characterized by very high levels of citric acid (CA, 245.60 and 222.14 mg/100 mL, respectively) and acetic acid (AA, 33.51 and 18.09 mg/100 mL). While high acetic acid can be detrimental, its presence in MJ5, combined with high levels of malic acid (MA, 233.43 mg/L) and succinic acid (SA, 6.71 mg/L), likely contributed to a sharp yet complex acidity that is often appreciated in certain rice wine styles (Liu et al., 2022). In contrast, MJ2 and MJ3 had much lower levels of most organic acids, which aligns with their lower total acidity and flat sensory profile. The exceptionally high tartaric acid (TA) in MJ7 (111.36 mg/L) and MJ6 (100.69 mg/L) is noteworthy, as TA is not a common microbial product but may be derived from raw materials or specific fermentation conditions (Yang et al., 2021). The data suggest that the interplay between different organic acids, rather than any single one, defines the sensory perception of sourness and overall taste balance (Cao et al., 2025).

**Table 3:** Organic acid contents of different fermented rice wines (mg/100 mL).

	MJ1	MJ2	MJ3	MJ4	MJ5	MJ6	MJ7
Oxalic acid (OA)	0.22 ± 0.01^e^	0.53 ± 0.01^c^	0.57 ± 0.01^b^	0.21 ± 0.01^e^	1.00 ± 0.02^a^	0.42 ± 0.01^d^	1.02 ± 0.04^a^
Tartaric acid (TA)	75.06 ± 0.43^e^	47.90 ± 2.42^g^	64.54 ± 0.87^f^	97.60 ± 0.48^c^	83.26 ± 0.10^d^	100.69 ± 0.42^b^	111.36 ± 0.11^a^
Malic acid (MA)	231.55 ± 0.94^b^	135.34 ± 1.04^f^	218.51 ± 0.85^c^	191.44 ± 1.83^d^	233.43 ± 1.87^b^	282.32 ± 0.71^a^	153.32 ± 0.69^e^
Lactic acid(LA)	50.71 ± 0.12^d^	63.62 ± 0.46^c^	66.67 ± 0.09^b^	74.27 ± 0.36^a^	32.45 ± 0.87^f^	40.98 ± 0.34^e^	67.10 ± 0.22^b^
Acetic acid (AA)	8.42 ± 0.05^c^	4.82 ± 0.12^f^	3.96 ± 0.06^g^	5.73 ± 0.12^e^	33.51 ± 0.82^a^	18.09 ± 0.60^b^	7.25 ± 0.05^d^
Citric acid (CA)	84.30 ± 0.15^f^	78.49 ± 1.02^g^	156.80 ± 1.50^e^	187.18 ± 1.89^d^	245.60 ± 2.22^a^	222.14 ± 1.50^b^	197.27 ± 0.41^c^
Succinic acid (SA)	4.78 ± 0.13^d^	1.72 ± 0.07^e^	1.91 ± 0.08^e^	7.61 ± 0.19^b^	6.71 ± 0.09^c^	7.85 ± 0.14^b^	11.82 ± 0.31^a^

Note: values with distinct superscript letters within the same row were statistically significant (*p* < 0.05).All findings are shown as the mean ± standard deviation (*n* = 3).

### 3.3 Biogenic amines and safety considerations

Biogenic amines (BAs) are nitrogenous compounds formed by the decarboxylation of amino acids by microorganisms. High levels of BAs, particularly histamine and tyramine, are of food safety concern.The total BA content varied widely, from 20.47 mg/L in MJ2 to a high of 132.54 mg/L in MJ6 (Table 4, Supplementary Table S5). Putrescine was the most abundant BA in most samples, followed by tyramine and cadaverine. MJ6 showed the highest levels of tyramine (57.38 mg/L) and tryptamine (6.15 mg/L). The high tyramine content in MJ6 is a potential safety concern, as it can cause hypertensive effects in sensitive individuals. This high BA level likely results from the activity of specific lactic acid bacteria (LAB) or other decarboxylase-positive bacteria present during fermentation (Luo et al., 2024; Yuan et al., 2025). In contrast, MJ2 had remarkably low levels of almost all BAs, particularly putrescine (7.59 mg/L) and tyramine (6.73 mg/L), indicating superior microbial control or a fermentation environment less conducive to BA formation.

**Table 4:** Biogenic amines contents of different fermented rice wines (mg/L).

	MJ1	MJ2	MJ3	MJ4	MJ5	MJ6	MJ7
Histamine	4.32 ± 0.05^f^	3.04 ± 0.07^g^	5.49 ± 0.09^e^	6.12 ± 0.06^d^	14.39 ± 0.12^a^	9.26 ± 0.21^b^	7.11 ± 0.14^c^
Putrescine	27.19 ± 0.39^d^	7.59 ± 0.05^f^	47.23 ± 1.02^a^	12.58 ± 0.07^e^	39.15 ± 1.20^c^	42.59 ± 0.78^b^	46.39 ± 0.86^a^
Cadaverine	1.32 ± 0.04^f^	0.61 ± 0.02^g^	4.53 ± 0.06^a^	2.05 ± 0.07^e^	4.17 ± 0.13^b^	3.79 ± 0.08^c^	2.76 ± 0.06^d^
Tryptamine	0.42 ± 0.02^g^	0.82 ± 0.01^e^	0.54 ± 0.01^f^	0.91 ± 0.03^d^	2.35 ± 0.05^b^	6.15 ± 0.10^a^	1.49 ± 0.04^c^
Tyramine	10.64 ± 0.31^e^	6.73 ± 0.03^f^	16.75 ± 0.07^b^	25.52 ± 0.11^c^	27.42 ± 0.16^b^	57.38 ± 0.74^a^	17.28 ± 0.12^d^
Spermidine	0.73 ± 0.01^e^	0.62 ± 0.01^f^	1.29 ± 0.02^b^	0.98 ± 0.03^c^	2.61 ± 0.02^a^	2.58 ± 0.04^a^	0.91 ± 0.02^d^
Spermine	2.38 ± 0.07^a^	0.34 ± 0.01^f^	0.82 ± 0.02^e^	1.65 ± 0.03^b^	1.27 ± 0.03^c^	2.43 ± 0.05^a^	0.94 ± 0.04^d^
Phenethylamine	1.14 ± 0.02^f^	0.72 ± 0.02^j^	3.78 ± 0.03^e^	5.34 ± 0.04^d^	6.45 ± 0.05^c^	8.36 ± 0.06^a^	7.49 ± 0.05^b^
Total	48.14 ± 0.72^f^	20.47 ± 0.13^j^	80.42 ± 1.14^d^	55.15 ± 0.08^e^	97.82 ± 1.32^b^	132.54 ± 1.66^a^	84.38 ± 0.69^c^

Note: values with distinct superscript letters within the same row were statistically significant (*p* < 0.05).All findings are shown as the mean ± standard deviation (*n* = 3).

Interestingly, MJ5, despite having high total FAA and high sensory scores, had moderate BA levels (97.82 mg/L), suggesting that its dominant microbial community was efficient in converting amino acids to alcohols and esters rather than to undesirable amines. This is consistent with findings that a well-managed fermentation with a balanced microbial ecosystem can reduce BA accumulation (Jiao et al., 2025; Yuan et al., 2025). The data highlight that while MJ6 and MJ7 have complex flavor profiles, their higher BA content necessitates careful process control to ensure safety. The low BAs in MJ2, while indicating safety, unfortunately coincide with a less flavorful product, demonstrating the challenge in balancing safety and sensory quality.

### 3.4 Volatile flavor compounds in different fermented rice wines

**Fig. 2:**
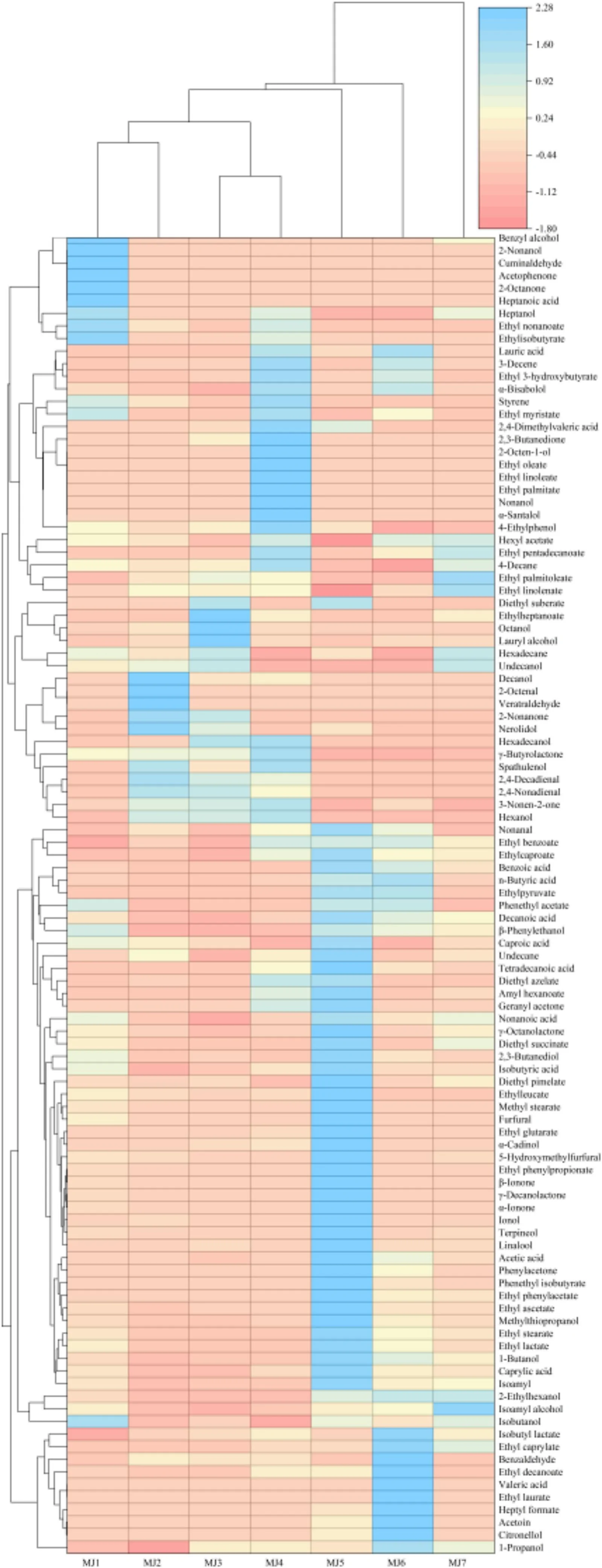
Clustered heatmap of volatile compounds across seven rice wine samples.

In total, 118 aroma compounds were identified in the seven rice wine samples: 29 alcohols, 47 esters, 19 ketones and aldehydes, 13 acids, 4 phenols, 2 alkenes, and 4 alkanes (Table 5, Supplementary Table S6). The types and numbers of these aroma components indicate that the major flavor contributors in fermented rice wine are alcohols, esters, ketones/aldehydes, and acids, with esters playing a particularly crucial role. The clustered heatmap revealed that the seven rice wine samples segregated into two major groups, with MJ5 and MJ6 forming a distinct cluster characterized by elevated levels of key esters (e.g., ethyl acetate, phenethyl acetate, ethyl lactate, and ethyl caprylate) and higher alcohols (e.g., β-phenylethanol and isoamyl alcohol), alongside unique floral compounds such as linalool, terpineol, and β-ionone in MJ5 (Fig. 2).

**Table 5:** Volatile compound composition and content in fermented rice wines (μg/L).

Flavor components	MJ1	MJ2	MJ3	MJ4	MJ5	MJ6	MJ7
Alcohols							
1-Propanol	22.03 ± 1.33e	9.78 ± 0.16f	38.1 ± 1.15c	38.87 ± 0.46c	35.65 ± 0.48d	64.65 ± 2.36a	45.03 ± 2.07b
Isobutanol	654.39 ± 11.01a	364.24 ± 27.15e	403.17 ± 31.2d	299.14 ± 19.91f	524.7 ± 19.6b	460.92 ± 19.55c	557.97 ± 9.51b
1-Butanol	11.4 ± 0.27c	6.82 ± 0.5d	7.68 ± 0.41d	7.41 ± 0.34d	25.6 ± 1.69a	12.49 ± 0.85c	14.07 ± 1.1b
Isoamyl alcohol	4731.32 ± 361.83a	2139.91 ± 69.64d	2323.63 ± 146.19d	2513.79 ± 103.79d	4993.27 ± 244.58a	4287.02 ± 354.1b	3833.57 ± 254.75c
Hexanol	ND	123.62 ± 6.62b	119.61 ± 11.34b	148.17 ± 2.06a	ND	ND	ND
Heptanol	13.82 ± 0.67c	20.98 ± 1.63b	19.7 ± 0.87b	21.94 ± 0.55b	ND	14.75 ± 1.02c	32.37 ± 1.99a
2-Ethylhexanol	50.06 ± 1.77b	10.84 ± 0.51d	16.63 ± 0.97c	14.45 ± 0.81c	55.62 ± 1.05a	55.41 ± 3.01a	11.33 ± 0.5d
2-Nonanol	4.58 ± 0.15c	6.04 ± 0.57b	8.87 ± 0.45a	ND	ND	ND	9.1 ± 0.68a
2,3-Butanediol	220.99 ± 2.74b	ND	51.77 ± 0.58f	145.71 ± 3.66c	373.69 ± 4.85a	98.86 ± 9.64e	109.11 ± 7.53d
Linalool	5.3 ± 0.34c	ND	15.77 ± 0.89b	4.33 ± 0.14c	227.96 ± 19.32a	17.14 ± 1.61b	10.65 ± 0.86b
Octanol	61.02 ± 4.11b	44.31 ± 1.84d	52.78 ± 3.26c	95.75 ± 2.28a	49.96 ± 1.85c	23.09 ± 1.89f	33 ± 2.04e
2-Octen-1-ol	4.52 ± 0.26a	ND	ND	2.48 ± 0.09a	ND	ND	ND
Nonanol	16.55 ± 0.51c	15.08 ± 1.14d	13.31 ± 0.71e	24.93 ± 0.45a	15.82 ± 0.37 cd	22.71 ± 1.12b	16.17 ± 0.21 cd
Terpineol	7.51 ± 0.3c	ND	ND	5.55 ± 0.36c	350.53 ± 6.04a	ND	16.76 ± 0.84b
Methylthiopropanol	11.44 ± 1.12c	ND	ND	8.26 ± 0.46c	41.22 ± 3.34a	14.45 ± 1.04b	11.22 ± 0.73c
Decanol	3.3 ± 0.27c	14.94 ± 0.5b	95.08 ± 7.89a	14.61 ± 0.39b	ND	12.41 ± 0.98b	16.52 ± 1.29b
Undecanol	ND	7.79 ± 0.36b	64.24 ± 4.1a	6.46 ± 0.21b	ND	ND	ND
Citronellol	ND	ND	ND	ND	9.47 ± 0.13	43.68 ± 2.22	ND
Benzyl alcohol	16.68 ± 1.54b	13.2 ± 1.2c	19.47 ± 0.59a	6.46 ± 0.53d	12.4 ± 0.5c	5.62 ± 0.49d	19.68 ± 1.21a
β-Phenylethanol	3592.63 ± 150.36d	2153.98 ± 146.21f	2495.68 ± 217.11f	2629.66 ± 63.29e	8456.95 ± 307.84a	5758.81 ± 233.54b	4354.49 ± 166.35c
Lauryl alcohol	84.6 ± 1.66c	185.9 ± 12.12b	1147.81 ± 104.76a	72.5 ± 6.49c	1142.32 ± 18.8a	35.87 ± 2.12d	15.24 ± 0.35d
Ionol	3.59 ± 0.18a	ND	ND	ND	129.57 ± 5.53	ND	ND
Hexadecanol	ND	33.11 ± 2.39a	18.11 ± 1.54b	ND	7.16 ± 0.16c	ND	ND
Nerolidol	ND	7.8 ± 0.49a	5.86 ± 0.43b	ND	ND	ND	ND
α-Cadinol	ND	ND	1.38 ± 0.06c	5.39 ± 0.34b	34.79 ± 1.79a	ND	ND
Spathulenol	27.59 ± 2.03c	34.43 ± 3.04b	32.65 ± 1.89b	54.66 ± 4.56a	ND	ND	5.41 ± 0.14d
α-Santalol	ND	1.18 ± 0.06c	11.66 ± 0.63b	13.33 ± 0.53a	ND	ND	ND
α-Bisabolol	8.82 ± 0.12	ND	ND	ND	ND	ND	ND
Esters	ND	ND	ND	ND	ND	ND	ND
Ethyl acetate	204.95 ± 3.77c	46.77 ± 2.03e	66.46 ± 4.4e	117.82 ± 0.78e	1619.27 ± 148.33a	459.97 ± 35.58b	252.82 ± 6.73c
Ethyl isobutyrate	1.76 ± 0.04	ND	ND	ND	ND	ND	ND
Isoamyl acetate	139.03 ± 6.81b	55.48 ± 4.94c	34.18 ± 2.82d	62.67 ± 5.28c	139.02 ± 4.73b	160.54 ± 8.65b	295.48 ± 28.57a
Ethyl caproate	26.25 ± 1.05e	48.45 ± 3.65d	ND	160.92 ± 7.36b	275.64 ± 8.79a	135.17 ± 9.26c	127.41 ± 8.48c
Ethyl heptanoate	ND	ND	ND	4.44 ± 0.43	ND	ND	ND
Ethyl pyruvate	ND	ND	ND	ND	18.4 ± 1.08a	17.57 ± 0.46a	ND
Ethyl lactate	216.04 ± 0.77c	ND	ND	ND	750.25 ± 11.94a	317.84 ± 11.02b	142.29 ± 1.07d
Ethyl caprylate	6.23 ± 0.19e	3.51 ± 0.33e	ND	82.02 ± 5.52d	95.55 ± 8.82c	477.35 ± 1.42a	257.71 ± 4.22b
Ethyl leucate	57.2 ± 2.72b	15.15 ± 1.11c	12.08 ± 1.08 cd	3.88 ± 0.08e	197.74 ± 6.66a	8.92 ± 0.71de	6.21 ± 0.15e
Heptyl formate	ND	ND	ND	ND	1.54 ± 0.05a	12.78 ± 0.8b	ND
Isobutyl lactate	ND	2.83 ± 0.27c	3.21 ± 0.23c	5.35 ± 0.22b	3.08 ± 0.19c	12.03 ± 1.07a	5.07 ± 0.14b
Amyl hexanoate	ND	ND	ND	13.85 ± 0.79a	23.1 ± 0.61b	ND	ND
Ethyl 3-hydroxybutyrate	5.84 ± 0.3	ND	ND	ND	ND	ND	ND
Ethyl nonanoate	6.73 ± 0.61a	ND	ND	ND	ND	ND	2.29 ± 0.17b
γ-Butyrolactone	9.47 ± 0.34e	28.48 ± 2.46c	32.87 ± 2.58b	37.58 ± 3.67a	ND	13.88 ± 0.69d	ND
Ethyl decanoate	22.18 ± 1.88c	ND	ND	78.76 ± 5.96b	82.62 ± 6.07b	293.91 ± 12.52a	5.49 ± 0.25d
Diethyl succinate	757.02 ± 32.27b	128.71 ± 6.01f	228.75 ± 0.79de	193.97 ± 2.89e	1407.65 ± 50.61a	426.16 ± 19.55c	244.44 ± 15.44d
Ethyl benzoate	ND	9.07 ± 0.83d	ND	12.47 ± 0.74c	26.01 ± 2.6a	15.15 ± 0.74b	1.42 ± 0.09e
Ethyl glutarate	ND	ND	ND	1.56 ± 0.14a	17.68 ± 1.01b	ND	ND
Ethyl phenylacetate	22.01 ± 1.23c	4.42 ± 0.39e	5.94 ± 0.23e	15.2 ± 0.46d	168.37 ± 3.16a	33.62 ± 2.05b	24.41 ± 1.39c
Phenethyl acetate	458.03 ± 3.99d	176.13 ± 10.96f	123.25 ± 4.98 g	326.94 ± 11.11e	1066.68 ± 28.51a	723.84 ± 27.31b	628.9 ± 20.51c
Ethyl laurate	ND	ND	ND	ND	ND	62.75 ± 5.9	ND
Ethyl phenylpropionate	ND	0.94 ± 0.04a	ND	ND	50.5 ± 1.46b	ND	ND
Phenethyl isobutyrate	ND	ND	ND	ND	15.19 ± 0.19a	3.99 ± 0.39b	2.55 ± 0.15c
Diethyl pimelate	6.02 ± 0.52b	1.82 ± 0.1 cd	0.96 ± 0.04d	2.32 ± 0.07c	20.62 ± 1.11a	ND	ND
Ethyl myristate	134.05 ± 5.77b	11.75 ± 1.05d	8.44 ± 0.72d	163.38 ± 9.73a	ND	76.96 ± 7.58c	3.91 ± 0.35d
γ-Octanolactone	3.29 ± 0.31c	ND	ND	ND	10.26 ± 0.91a	ND	4.83 ± 0.46b
Diethyl suberate	ND	ND	ND	5.73 ± 0.51	ND	ND	ND
Ethyl pentadecanoate	ND	ND	ND	2.66 ± 0.12	ND	ND	ND
γ-Decanolactone	9.37 ± 0.46a	ND	ND	ND	154.94 ± 3.6b	ND	ND
Diethyl azelate	ND	ND	ND	15.52 ± 1.28b	28.85 ± 1.24a	ND	2.78 ± 0.16c
Ethyl palmitate	12.52 ± 0.43d	18.34 ± 1.22d	35.81 ± 2.95 cd	621.93 ± 45.63a	53.99 ± 5.4c	399.18 ± 8.86b	15.98 ± 0.5d
Ethyl palmitoleate	ND	ND	ND	8.67 ± 0.72	ND	ND	ND
Hexyl acetate	ND	ND	1.91 ± 0.05a	7.59 ± 0.38b	ND	ND	ND
Methyl stearate	7.45 ± 0.39b	4.95 ± 0.29bc	2.58 ± 0.24c	ND	52.94 ± 3.8a	ND	5.83 ± 0.43bc
Ethyl stearate	64.1 ± 3.08c	6.41 ± 0.41e	5.61 ± 0.46e	12.87 ± 0.21d	282.08 ± 3.48a	113.64 ± 6.35b	65.16 ± 5.07c
Ethyl oleate	ND	3.41 ± 0.1c	4.92 ± 0.37c	167.7 ± 8.75a	25.54 ± 1.36d	171.75 ± 9.23a	10.53 ± 0.8c
Ethyl linoleate	ND	5.49 ± 0.27c	3.75 ± 0.29c	221.74 ± 2.46a	ND	158.36 ± 10.13b	5.74 ± 0.45c
Ethyl linolenate	ND	ND	ND	7.64 ± 0.61	ND	ND	ND

Ketones							
2-Octanone	92.31 ± 6.49b	86.67 ± 4.76b	89.77 ± 6.87b	108.52 ± 4.25a	75.7 ± 6.74c	69.36 ± 3.9c	97.54 ± 6.53b
Acetoin	ND	ND	ND	ND	117.8 ± 10.59a	583.47 ± 16.37b	ND
2-Nonanone	ND	9.44 ± 0.74	ND	ND	ND	ND	ND
3-Nonen-2-one	ND	5.4 ± 0.5a	5.09 ± 0.35a	2.97 ± 0.11b	ND	ND	ND
Acetophenone	ND	ND	ND	5.94 ± 0.21a	ND	2.11 ± 0.2c	4.9 ± 0.24b
2,3-Butanedione	14.85 ± 0.17a	3.95 ± 0.26c	ND	9.82 ± 0.53b	ND	ND	ND
Phenylacetone	ND	ND	ND	ND	15.57 ± 0.55b	5.19 ± 0.21	ND
β-Ionone	73.62 ± 3.68a	ND	ND	ND	973.66 ± 19.92b	ND	ND
Geranyl acetone	31.94 ± 0.16b	9.96 ± 0.9e	ND	14.25 ± 0.84d	50.28 ± 3.71a	20.16 ± 1.94c	32.93 ± 3.07b
α-Ionone	69.11 ± 5.25a	ND	ND	ND	1542.16 ± 45.7b	ND	ND

Aldehydes							
Nonanal	13.6 ± 0.42d	21.07 ± 1.49c	15.86 ± 1.26d	32.74 ± 1.79a	33.75 ± 2.49a	33.17 ± 1.55a	26.63 ± 0.87b
2-Octenal	ND	13.41 ± 0.74a	2.77 ± 0.25b	2.97 ± 0.11b	ND	ND	ND
Furfural	5.67 ± 0.1c	5.12 ± 0.44c	4.52 ± 0.03c	5.97 ± 0.49c	182.96 ± 6.74a	31.37 ± 1.68b	ND
Benzaldehyde	ND	74.99 ± 7.41b	55.29 ± 3.16c	68.94 ± 5.87c	ND	268.03 ± 12.66a	ND
2,4-Nonadienal	ND	10.18 ± 0.5a	7.98 ± 0.79b	5.81 ± 0.21c	ND	ND	ND
2,4-Decadienal	ND	6.17 ± 0.5a	2.21 ± 0.16b	6.9 ± 0.68c	ND	ND	ND
Veratraldehyde	ND	736.26 ± 63.06	ND	ND	ND	ND	ND
Cuminaldehyde	7.29 ± 0.37bc	6.47 ± 0.54c	5 ± 0.14d	8.61 ± 0.15a	3.51 ± 0.13e	8.06 ± 0.79ab	8.4 ± 0.75a
5-Hydroxymethylfurfural	7.92 ± 0.12a	ND	ND	ND	51.79 ± 5.08b	ND	ND

Acids							
Acetic acid	64.22 ± 2.12c	48.52 ± 1.32d	32.76 ± 2.88e	53.82 ± 3.49d	317.33 ± 22.91a	156.96 ± 11.02b	68.24 ± 4.12c
Isobutyric acid	26.87 ± 1.66c	22 ± 2d	24.66 ± 0.94c	12.82 ± 0.86e	78.65 ± 7.09a	20.31 ± 1.4d	33.09 ± 1.59b
n-Butyric acid	4.59 ± 0.4c	3.9 ± 0.34d	3.55 ± 0.22e	5.12 ± 0.4c	11.75 ± 1b	13.57 ± 0.58a	4.76 ± 0.39c
2,4-Dimethylvaleric acid	36.74 ± 1.26a	8.19 ± 0.07d	7.27 ± 0.26d	25.83 ± 1.54b	ND	ND	23.28 ± 1.9c
Valeric acid	ND	ND	ND	ND	ND	135.79 ± 3.03	ND
Caproic acid	98.27 ± 5.26d	215.51 ± 6.27b	ND	188.71 ± 11.13c	471.57 ± 3.34a	85.96 ± 4.75d	174.59 ± 17.01c
Heptanoic acid	ND	1.53 ± 0.13d	3.13 ± 0.14b	2.5 ± 0.22c	ND	ND	5.58 ± 0.3a
Caprylic acid	349.19 ± 20.32c	101.65 ± 0.95d	100.55 ± 4.41d	58.61 ± 3.17d	732.06 ± 14.71b	913.19 ± 24.4a	897.52 ± 37.57a
Nonanoic acid	31.35 ± 0.74b	23.04 ± 2.26c	16.54 ± 1.33d	22.87 ± 2.27c	58.57 ± 2.53a	23.5 ± 1.92c	31.96 ± 1.15b
Decanoic acid	73.54 ± 0.91d	23.74 ± 2.17e	31.50 ± 1.96e	33.73 ± 0.98e	741.08 ± 31.98a	522.05 ± 32.56b	190.15 ± 8.25c
Benzoic acid	12.72 ± 1.1b	8.47 ± 0.54c	5.56 ± 0.29d	ND	21.58 ± 1.62a	ND	6.41 ± 0.42d
Lauric acid	8 ± 0.33	ND	ND	ND	ND	ND	ND
Tetradecanoic acid	ND	3.07 ± 0.15c	ND	22.47 ± 1.78b	26.98 ± 2.55a	ND	ND
4-Ethylphenol	16.28 ± 0.8d	16.03 ± 1.49d	32.91 ± 1.73c	181.85 ± 6.8a	109.23 ± 2.98b	ND	ND

Olefins							
3-Decene	11.08 ± 0.94	ND	ND	ND	ND	ND	ND
Styrene	16.39 ± 0.58b	5.82 ± 0.56c	ND	26.97 ± 1.39a	ND	ND	ND

Alkanes							
4-Decane	ND	ND	ND	23.57 ± 1.74	ND	ND	ND
Undecane	ND	ND	ND	5.57 ± 0.31b	17.28 ± 0.77a	2.81 ± 0.16c	0.76 ± 0.04d
Hexadecane	ND	ND	7.32 ± 0.4a	1.93 ± 0.04b	ND	ND	1.69 ± 0.07b

Note: values with distinct lowercase characters within the same row were statistically significant (*p* < 0.05). All findings are shown as the mean ± standard deviation (*n* = 3). ND, not detected.

In contrast, MJ2 and MJ3 clustered closely together, showing higher relative abundances of certain aldehydes (e.g., benzaldehyde, veratraldehyde, and 2,4-nonadienal) and medium-chain fatty acids, while MJ1, MJ4, and MJ7 occupied intermediate positions with mixed profiles (Fig. 2). These distribution patterns corroborate the sensory evaluation results, confirming that ester- and alcohol-rich volatile profiles are the primary drivers of superior sensory quality, whereas samples with lower volatile diversity and abundance exhibited plainer sensory characteristics.

Higher alcohols contribute to both pleasant (fruity, floral) and harsh (alcoholic) notes. β-Phenylethanol, which imparts a rose-like fragrance, was exceptionally high in MJ5 (8456.95 μg/L) and MJ6 (5758.81 μg/L), which correlates strongly with their high sensory scores (93.6 and 91.4, respectively). This compound is a key contributor to the desirable floral aroma in rice wine (Cui et al., 2024; Yu et al., 2019). The presence of linalool (227.96 μg/L) and terpineol (350.53 μg/L) only in MJ5 provides a unique floral and citrus dimension, likely contributing to its top sensory rating. In contrast, MJ2 and MJ3 showed much lower levels of these beneficial alcohols. Isoamyl alcohol, which can be malty or harsh depending on concentration, was highest in MJ5 (4993.27 μg/L) and MJ1 (4731.32 μg/L). While high in MJ5, its effect was likely balanced by the high ester content, whereas in MJ1, it may have contributed to a more pungent aroma, explaining its moderate sensory score (83.4) despite relatively high alcohol (Chen et al., 2013).

Esters are the primary contributors to fruity aromas and are highly desired in rice wine (Yang, Li, et al., 2024). MJ5 and MJ6 again stood out. Ethyl acetate, which provides a pineapple-like note, was dramatically higher in MJ5 (1619.27 μg/L) compared to other samples (e.g., 204.95 μg/L in MJ1). Furthermore, phenethyl acetate (honey, rose) was also highest in MJ5 (1066.68 μg/L), which, together with β-phenylethanol, explains the intense floral and fruity character of this sample. Diethyl succinate, which imparts a fruity and wine-like aroma, was most abundant in MJ5 (1407.65 μg/L) and MJ1 (757.02 μg/L). These results are consistent with studies showing that co-fermentation or specific starter cultures can significantly boost ester production (Cui et al., 2024; He et al., 2025). The presence of unique esters like γ-decanolactone in MJ5 (154.94 μg/L) and MJ1 (9.37 μg/L) adds a peachy, coconut-like nuance, further enhancing flavor complexity.

While volatile acids can contribute to rancid notes, they also serve as precursors for esters. Acetic acid was highest in MJ5 (317.33 μg/L), which, if present in excess, could be a defect. However, its high concentration in MJ5 was apparently balanced by the high ester and alcohol content, resulting in a well-integrated aroma. Caprylic acid, which has a slightly oily and rancid note, was exceptionally high in MJ6 (913.19 μg/L) and MJ7 (897.52 μg/L). This may explain why, despite having high FAAs and some beneficial alcohols, MJ7's sensory score (88.9) did not surpass MJ5's, as high levels of certain fatty acids can be perceived as off-flavors (Shen et al., 2024). The presence of β-ionone (973.66 μg/L) and α-ionone (1542.16 μg/L), which have violet and woody aromas, exclusively in MJ5 provides a unique and highly desirable floral complexity, solidifying its position as the highest-quality sample in this study (Chen et al., 2018).The volatile profiles clearly distinguished the seven samples in terms of chemical diversity and abundance, with the highest ester and higher alcohol contents observed in MJ5 and MJ6, aligning with their superior sensory ratings, while MJ2 and MJ3 exhibited notably lower volatile complexity (Table 5).

### 3.5 Implications for quality standardization

The integrated analysis presented in this study offers a practical roadmap for establishing comprehensive quality standards for fermented rice wines. First, we propose that a core set of mandatory indicators should include alcohol content (reflecting fermentation completeness), total acidity (influencing microbial stability and taste), amino acid nitrogen (contributing to flavor and nutrition), key esters (e.g., ethyl acetate and phenethyl acetate, which drive consumer preference), and total biogenic amines (ensuring safety). Second, our data suggest a provisional safety threshold of <100 mg/L for total biogenic amines, as samples exceeding this level (e.g., MJ6 at 132.54 mg/L) raised legitimate safety concerns, while the highest-quality sample (MJ5) remained below this limit. Third, sensory-chemical correlations enable quality grading: premium wines (> 90 points) are characterized by high ester contents (> 2000 μg/L) and elevated umami amino acids, whereas plain wines (< 85 points) exhibit low volatile diversity and limited amino acid complexity. Fourth, regional benchmarking is essential, as local raw materials and starter cultures produce distinct profiles; standards should therefore be adaptable while maintaining core safety and sensory criteria. Finally, our findings guide process optimization—promoting ester formation through selected starter cultures and fermentation conditions, while controlling biogenic amine accumulation via microbial management. These recommendations provide an empirical foundation that can support future regulatory efforts and industrial quality improvement.

### 3.6 Limitations and future perspectives

It should be noted that although a uniform storage protocol was applied to all samples (−18 °C before analysis and 4 °C after opening, with measurements completed within 7 days), the stability of individual quality indicators under these conditions was not experimentally verified. Freezing does not fully inactivate enzymes (e.g., lipases, esterases, and proteases) or suppress psychrotolerant microbial activity, which may theoretically cause subtle changes over time, including ester hydrolysis, organic acid interconversion, or decarboxylation of amino acids to biogenic amines (Luo et al., 2024; Yuan et al., 2025). Moreover, volatile compounds with low boiling points or high hydrophobicity (e.g., ethyl acetate and isoamyl alcohol) may partition into the headspace and be lost during thawing or repeated pipetting (Wang et al., 2013). Consequently, the absolute concentrations in Table 5 may not fully reflect the original fresh-state profiles. Nevertheless, since all samples were subjected to identical storage duration, temperature, and handling, the relative comparisons and quality rankings remain robust; any storage-induced deviations would have affected all groups uniformly. Future studies should include systematic stability tests under various storage conditions to validate absolute quantitation.

Additionally, this study analyzed only a single batch per manufacturer and did not assess batch-to-batch reproducibility. The findings thus represent a snapshot of product quality at the time of collection (March 2025), rather than consistency across production cycles. Traditional rice wine fermentation, conducted in open or semi-open systems, is inherently variable due to differences in raw materials, starter cultures, fermentation conditions, and post-fermentation storage. Moreover, as noted in Section 2.1, specific production parameters were not disclosed for proprietary reasons, precluding a systematic evaluation of their influence on reproducibility. Future work should incorporate multiple batches from the same manufacturer to identify critical control points affecting consistency. The multi-parameter analytical framework established here—covering physicochemical indices, amino acids, organic acids, biogenic amines, volatiles, and sensory evaluation—provides a standardized platform for such investigations.

## 4 Conclusion

This study confirms that the quality of fermented rice wine is not governed by any single physicochemical or flavor parameter, but rather by the intricate synergy among volatile compounds (particularly esters and higher alcohols), non-volatile taste components (amino and organic acids), and the critical safety index of biogenic amines. Among the seven commercial samples evaluated, MJ5 (Guangxi) emerged as the benchmark for optimal flavor balance, whereas MJ6 (Hubei) exemplified the challenge of managing flavor complexity alongside BA safety risks, and MJ2 (Chongqing) illustrated the trade-off between safety and sensory richness.

These findings underscore the necessity for a comprehensive, multi-target quality assurance framework for the Chinese rice wine industry. Future production improvements should prioritize the selection of starter cultures and fermentation conditions that enhance desirable ester synthesis and controlled proteolysis while actively suppressing the decarboxylase activity of spoilage microorganisms to limit BA accumulation. This study provides a scientific reference for standardizing regional rice wine quality and fostering consumer confidence in traditional fermented beverages.

## Studies in human

This study was performed in compliance with relevant laws, regulatory frameworks and guidelines where the research took place.

The Science and Technology Ethics Committee of Yibin University granted an exemption. The authors provided the following explanation: It does not involve any clinical intervention, collection of personal health information, or experimentation on human subjects.

## CRediT authorship contribution statement

**HuaWei Yuan:** Writing – original draft, Software, Methodology, Investigation, Funding acquisition, Data curation, Conceptualization. **XinYu Zhao:** Validation, Investigation. **Hao Yuan:** Resources, Methodology. **ZiYing Tian:** Methodology, Investigation. **Zhou Xu:** Investigation, Funding acquisition. **TingNa Xie:** Validation, Funding acquisition, Formal analysis. **Kai Lou:** Writing – review & editing, Supervision, Project administration, Conceptualization.

## Informed consent and patient details

Written informed consent to take part in the study and to publish the article has been obtained from all participants or their legal representatives. The privacy rights of participants have been observed.

## Ethics statement

Prior to the sensory tests, panelists from Yibin University (Yibin, China) were fully informed of the test details, requirements, and risks, and signed informed consent forms. The study was authorized by the Science and Technology Ethics Committee of Yibin University for sensory evaluation. Participants' rights and privacy were protected through voluntary participation, full disclosure, informed consent, data confidentiality, and the right to withdraw at any time.

## Declaration of competing interest

The authors declare that they have no known competing financial interests or personal relationships that could have appeared to influence the work reported in this paper.

## Data Availability

Data will be made available on request.

## References

[bb0005] Cao X., Zhang Q., Wang W., Wang L., Wang J., Ma Y., Zhao Q. (2025). Influence of different glutinous rice ratios on flavor quality of rice wine with jujube. Food Research and Development.

[bb0010] Cen L., Shi X., Zhang L., Qiu S., Zeng X., Dai Y., Wei C. (2023). Effects of *Dendrobium officinale* on the quality of rice wine fermented separately by *Saccharomyces cerevisiae* and *Wickerhamomyces anomalus*: Physicochemical indices, volatile compounds and nonvolatile metabolites. Fermentation.

[bb0015] Chen C., Liu Y., Tian H., Ai L., Yu H. (2020). Metagenomic analysis reveals the impact of JIUYAO microbial diversity on fermentation and the volatile profile of Shaoxing-jiu. Food Microbiology.

[bb0020] Chen S., Wang D., Xu Y. (2013). Characterization of odor-active compounds in sweet-type Chinese rice wine by aroma extract dilution analysis with special emphasis on sotolon. Journal of Agricultural and Food Chemistry.

[bb0025] Chen S., Xu Y., Qian M.C. (2018). Comparison of the aromatic profile of traditional and modern types of Huang jiu (Chinese rice wine) by aroma extract dilution analysis and chemical analysis. Flavour and Fragrance Journal.

[bb0030] Cui R., Liu X., Wang Q., Wang S., He J., Zhou S., Yao Y. (2024). Effects of co-fermentation of high-yield ester *Zygosaccharomyces rouxii* and *Saccharomyces cerevisiae* on the quality of rice wine. JSFA Reports.

[bb0035] Deng Y., Zheng H., Zhao M., Cao C., Kan H., Wu B., Liu Y. (2025). Flavor characteristics and metabolomics of sweet rice wine fermented with different non-*Saccharomyces* yeasts. Food Research International.

[bb0040] General Administration of Quality Supervision, Inspection and Quarantine of China, Standardization Administration of China (2006).

[bb0050] He Y., Feng Y., Wu H., Qin Y., Lu Y., Gao Z., Fan F. (2025). Effects of synergistic fermentation of yeast in Yunnan rice wine and *Rhizopus oryzae* on physicochemical properties and flavor substances of rice wine. Science and Technology of Food Industry.

[bb0055] He Y., Yuan G., Wang C. (2022). Correlation analysis between starch and glucose content in rice wine brewing. Food and Fermentation Industries.

[bb0060] Huang Y., Yin L., Xu W., Duan C., Ma F., Ma Y., Li D. (2023). Antioxidant capacity and flavor compounds of lily rice wine fermented with aroma-producing yeasts. European Food Research and Technology.

[bb0065] International Organization for Standardization (1994).

[bb0070] Jiao S., Kou Y., Cai W., Xiong Y., Fan H., Fu G., Wan Y. (2025). Impact of *Wickerhamomyces anomalus* enhancement on quality of liquid-fermented Hongqu rice wine. Food and Fermentation Industries.

[bb0075] Koay M., Fan H.Y., Wong C.M.V.L. (2024). Fermentation dynamics, physicochemical and sensory characteristics of Malaysian traditional rice wines. British Food Journal.

[bb0080] Lin X., Ren X., Huang Y., Liang Z., Li W., Su H., He Z. (2021). Regional characteristics and discrimination of the fermentation starter HongQu in traditional rice wine brewing. International Journal of Food Science & Technology.

[bb0085] Liu A., Yang X., Guo Q., Zheng Y., Shi Y., Zhu L., Li B. (2022). Microbial communities and flavor compounds during the fermentation of traditional Hong Qu glutinous rice wine. Foods.

[bb0090] Liu Q., Chen Y., Li S., Zhen D. (2025). Optimization of fermentation process and volatile flavor compounds of selenium-rich black glutinous rice wine. China Brewing.

[bb0095] Luo Y., Zhang C., Liao H., Luo Y., Huang X., Wang Z., Xia X. (2024). Integrative metagenomics, volatilomics and chemometrics for deciphering the microbial structure and core metabolic network during Chinese rice wine (Huangjiu) fermentation in different regions. Food Microbiology.

[bb0100] National Health Commission of China, Standardization Administration of China (2016).

[bb0105] National Health Commission of China, Standardization Administration of China (2016).

[bb0110] Peng B., Huang H., Xu J., Xin Y., Hu L., Wen L., Li C. (2025). Rice wine fermentation: Unveiling key factors shaping quality, flavor, and technological evolution. Foods.

[bb0115] Qian M., Ruan F., Zhao W., Dong H., Bai W., Li X., Li Y. (2023). The dynamics of physicochemical properties, microbial community, and flavor metabolites during the fermentation of semi-dry Hakka rice wine and traditional sweet rice wine. Food Chemistry.

[bb0120] Shen C., Yu Y., Zhang X., Zhang H., Chu M., Yuan B., Xu X. (2024). The dynamic of physicochemical properties, volatile compounds and microbial community during the fermentation of Chinese rice wine with diverse cereals. Food Research International.

[bb0125] Shi X., Zhang L., Chen H., Fu P., Zhang W., Qiu S., Wei C. (2022). Analysis of quality of *Dendrobium candidum* rice wine fermented by mixed yeast of *Saccharomyces cerevisiae* and *Wickerhamomyces anomalus*. Food and Fermentation Science & Technology.

[bb0135] Tang A., Peng B. (2024). Metatranscriptomics reveals microbial community function succession and characteristic flavor formation mechanisms during black rice wine fermentation. Food Chemistry.

[bb0140] Wang D., Jin B., Xu Y., Zhao G. (2013). Sensory flavor characteristics of Chinese yellow rice wine and construction of flavor wheel. Food Science.

[bb0145] Wong B., Owens A., Phillips M., Kam R. (2023). Identifying sensory attributes of Korean rice wine (makgeolli) using sensory evaluation and chemical analysis. Journal of Food Science.

[bb0150] Xiao C., Wang L., Zhang Y.-G., Tu T.-Y., Wang S.-T., Shen C.-H., Zhong X.-Z. (2021). A comparison of microbial communities and volatile compounds in wheat *Qu* from different geographic locations. LWT- Food Science and Technology.

[bb0155] Yan Q., Wu W., Zhang K. (2021). Correlation among volatile flavor substances, amino acids and sensory evaluation of Mimaiqu rice wine. China Brewing.

[bb0160] Yang C., Shen X., Ma X. (2018). Determination of organic acids in rice wine by high performance liquid chromatography. Food Research and Development.

[bb0165] Yang Y., Li H., Xia Y., Li S., Wang G., Ni L., Ai L. (2024). Unraveling physicochemical characteristics, microbial community structure and functional enzymes governing fatty acid ethyl esters formation during Chinese rice wine brewing. LWT- Food Science and Technology.

[bb0170] Yang Y., Xia Y., Hu W., Tao L., Liu H., Xie C., Ai L. (2021). Soaking induced discrepancies in oenological properties, flavor profiles, microbial community and sensory characteristic of Huangjiu (Chinese rice wine). LWT- Food Science and Technology.

[bb0175] Yang Y., Xia Y., Wang G., Hu W., Tao L., Liu H., Xie C., Ai L. (2017). Comparison of oenological property, volatile profile, and sensory characteristic of Chinese rice wine fermented by different starters during brewing. International Journal of Food Properties.

[bb0180] Yang Y., Xia Y., Wang G., Song X., Ni L., Ai L. (2024). Screening appropriate lactic acid bacteria as adjunct starter to enhance characteristic flavor and sensory profiles of Huangjiu (Chinese rice wine). Food Bioscience.

[bb0185] Yang Y., Xia Y., Wang G., Yu J., Ai L. (2017). Effect of mixed yeast starter on volatile flavor compounds in Chinese rice wine during different brewing stages. LWT- Food Science and Technology.

[bb0190] Yu H., Guo W., Xie J., Ai L., Chen C., Tian H. (2022). Evaluation of taste characteristics of Chinese rice wine by quantitative description analysis, dynamic description sensory and electronic tongue. Journal of Food Measurement and Characterization.

[bb0195] Yu H., Xie T., Xie J., Ai L., Tian H. (2019). Characterization of key aroma compounds in Chinese rice wine using gas chromatography-mass spectrometry and gas chromatography-olfactometry. Food Chemistry.

[bb0200] Yuan H., Zhang C., Chen S., Zhao Y., Tie Y., Yin L. (2022). Effect of different moulds on oenological properties and flavor characteristics in rice wine. LWT- Food Science and Technology.

[bb0205] Yuan Y., Lei H., Liang Z., Hou S., Luo Y., Jiang M., Ni L. (2025). Effects of enhanced fermentation with *Saccharomyces cerevisiae* on the microbial composition and the flavor quality in Hongqu rice wine. Journal of Chinese Institute of Food Science and Technology.

[bb0210] Zhang Z., Li Y., Bai L., Chen P., Jiang Y., Qi Y., Yan G. (2024). Machine learning combined with multi-source data fusion for rapid quality assessment of yellow rice wine with different aging years. Microchemical Journal.

[bb0215] Zhao C., Su W., Mu Y., Jiang L., Mu Y. (2020). Correlations between microbiota with physicochemical properties and volatile flavor components in black glutinous rice wine fermentation. Food Research International.

[bb0220] Zhao C., Su W., Mu Y., Luo L., Zhao M., Qiu S., Jiang L. (2023). Effects of Jiuqu inoculating *Rhizopus oryzae* Q303 and *Saccharomyces cerevisiae* on chemical components and microbiota during black glutinous rice wine fermentation. International Journal of Food Microbiology.

