# Corrigendum to “Quality characterization of fermented rice wines: A multivariate study on physicochemical indices, biogenic amines, volatile profiles, and sensory evaluation” [Food Chem.: X 38 (2026) 104364]

**DOI:** 10.1016/j.fochx.2026.104610

**Published:** 2026-10-19

**Authors:** HuaWei Yuan, XinYu Zhao, Hao Yuan, ZiYing Tian, Zhou Xu, TingNa Xie, Kai Lou

**Affiliations:** Faculty of Quality Management and Inspection & Quarantine / Solid-State Fermentation Resource Utilization Key Laboratory of Sichuan Province / Engineering Research Center of Agri-food Standardization and Inspection of The Education Department of Sichuan Province, Yibin University, Yibin, Sichuan, China

The authors regret that several inadvertent errors occurred in the original publication.(See Table 1, Table 2, Table 3, Table 4, Table 5.)

**Table 1:** The physicochemical parameters and sensory scores of the seven rice wine samples.

	MJ1	MJ2	MJ3	MJ4	MJ5	MJ6	MJ7
Total Acidity (g/L)	3.23±0.16^c^	1.90±0.09^e^	1.99±0.01^e^	2.74±0.09^d^	5.53±0.15^a^	5.02±0.23^b^	2.51±0.08^d^
Volatile acid (g/L)	0.18±0.01^f^	0.23±0.01^e^	0.24±0.01^e^	0.50±0.01^b^	0.67±0.02^a^	0.43±0.01^c^	0.32±0.02^d^
Total sugar (g/L)	96.84±0.13^g^	171.07±0.91^c^	159.19±0.32^d^	188.89±0.29^b^	137.29±0.19^e^	209.75±0.47^a^	127.80±0.82^f^
Reducing Sugar (g/L)	85.28±0.18^e^	160.78±0.61^a^	112.85±0.62^b^	108.62±0.56^c^	71.29±0.64^f^	109.04±1.01^c^	87.19±0.89^d^
Amino acid nitrogen (mg/100mL)	12.13±0.20^e^	20.80±0.59^c^	24.53±0.74^b^	20.59±0.43^c^	27.13±0.40^a^	14.72±0.02^d^	11.49±0.39^e^
Alcohol Content (%vol)	6.81±0.02^b^	4.45±0.01^e^	4.43±0.02^e^	5.42±0.03^d^	8.35±0.03^a^	5.43±0.01^d^	6.14±0.03^c^
Sensory score (points)	83.4±2.2^d^	88.8±2.4^c^	81.4±2.6^e^	80.3±2.4^e^	93.6±1.8^a^	91.4±2.5^b^	88.9±1.4^c^

Note: values with distinct superscript letters within the same row were statistically significant (*p* < 0.05). All findings are shown as the mean ± standard deviation (*n* = 3).

**Table 2:** Amino acid contents of different fermented rice wines (mg/100mL).

	MJ1	MJ2	MJ3	MJ4	MJ5	MJ6	MJ7
Asp	3.81±0.02^e^	6.41±0.02^d^	6.45±0.16^d^	6.35±0.07^d^	8.98±0.09^b^	6.82±0.03^c^	9.15±0.14^a^
Thr	1.62±0.03^e^	2.40±0.05^cd^	2.35±0.01^d^	2.43±0.03^c^	3.48±0.01^a^	2.70±0.02^b^	3.54±0.06^a^
Ser	2.18±0.03^f^	3.37±0.02^d^	3.24±0.03^e^	3.36±0.02^d^	4.87±0.02^b^	3.81±0.03^c^	5.09±0.05^a^
Glu	8.25±0.04^e^	12.48±0.05^d^	12.49±0.03^d^	12.40±0.09^d^	19.08±0.05^b^	14.06±0.14^c^	19.40±0.04^a^
Gly	2.43±0.03^e^	3.16±0.01^d^	3.16±0.01^d^	3.28±0.03^d^	5.05±0.01^b^	3.64±0.03^c^	7.32±0.11^a^
Ala	2.39±0.09^e^	3.64±0.02^cd^	3.60±0.02^d^	3.83±0.02^c^	5.84±0.03^b^	3.75±0.04^cd^	6.56±0.26^a^
Cys	0.57±0.02^d^	0.56±0.01^de^	0.51±0.01^f^	0.53±0.01^ef^	1.15±0.02^b^	0.76±0.02^c^	1.22±0.03^a^
Val	1.95±0.04^e^	3.53±0.05^d^	3.73±0.03^c^	3.60±0.02^d^	4.40±0.06^b^	3.48±0.16^d^	4.76±0.05^a^
Met	0.15±0.01^e^	0.23±0.01^d^	0.23±0.01^d^	0.23±0.01^d^	0.40±0.02^b^	0.30±0.01^c^	0.75±0.04^a^
Ile	1.40±0.03^e^	2.56±0.03^c^	2.72±0.06^b^	2.65±0.03^bc^	3.04±0.02^a^	2.36±0.11^d^	3.11±0.07^a^
Leu	2.33±0.03^f^	4.44±0.03^e^	4.51±0.02^d^	4.65±0.04^c^	5.05±0.04^b^	4.41±0.02^e^	5.13±0.04^a^
Tyr	1.14±0.02^f^	1.55±0.01^c^	1.55±0.02^c^	1.37±0.05^d^	2.22±0.01^b^	1.26±0.06^e^	2.33±0.03^a^
Phe	1.69±0.02^e^	3.28±0.02^d^	3.25±0.04^d^	3.27±0.03^d^	3.77±0.03^b^	3.50±0.08^c^	4.03±0.07^a^
Lys	1.83±0.03^f^	2.74±0.05^e^	2.83±0.08^d^	3.00±0.02^c^	3.69±0.05^b^	2.96±0.06^c^	3.89±0.02^a^
His	1.25±0.02^e^	1.63±0.02^d^	1.74±0.06^c^	1.73±0.04^c^	3.38±0.01^a^	2.07±0.03^b^	3.42±0.05^a^
Arg	3.92±0.08^f^	6.60±0.03^e^	6.77±0.04^d^	6.65±0.06^e^	9.07±0.02^b^	7.30±0.07^c^	9.71±0.03^a^
Pro	2.53±0.09^e^	3.08±0.02^d^	3.02±0.07^d^	3.07±0.02^d^	5.05±0.06^b^	3.62±0.02^c^	5.83±0.02^a^
Total	39.44±0.14^e^	61.64±0.33^d^	62.15±0.44^d^	62.42±0.51^d^	88.53±0.41^b^	66.81±0.73^c^	95.23±0.53^a^

Note: values with distinct superscript letters within the same row were statistically significant (*p* < 0.05).All findings are shown as the mean ± standard deviation (*n* = 3).

**Table 3:** Organic acid contents of different fermented rice wines (mg/100mL).

	MJ1	MJ2	MJ3	MJ4	MJ5	MJ6	MJ7
Oxalic acid (OA)	0.22±0.01^e^	0.53±0.01^c^	0.57±0.01^b^	0.21±0.01^e^	1.00±0.02^a^	0.42±0.01^d^	1.02±0.04^a^
Tartaric acid (TA)	75.06±0.43^e^	47.90±2.42^g^	64.54±0.87^f^	97.60±0.48^c^	83.26±0.10^d^	100.69±0.42^b^	111.36±0.11^a^
Malic acid (MA)	231.55±0.94^b^	135.34±1.04^f^	218.51±0.85^c^	191.44±1.83^d^	233.43±1.87^b^	282.32±0.71^a^	153.32±0.69^e^
Lactic acid(LA)	50.71±0.12^d^	63.62±0.46^c^	66.67±0.09^b^	74.27±0.36^a^	32.45±0.87^f^	40.98±0.34^e^	67.10±0.22^b^
Acetic acid (AA)	8.42±0.05^c^	4.82±0.12^f^	3.96±0.06^g^	5.73±0.12^e^	33.51±0.82^a^	18.09±0.60^b^	7.25±0.05^d^
Citric acid (CA)	84.30±0.15^f^	78.49±1.02^g^	156.80±1.50^e^	187.18±1.89^d^	245.60±2.22^a^	222.14±1.50^b^	197.27±0.41^c^
Succinic acid (SA)	4.78±0.13^d^	1.72±0.07^e^	1.91±0.08^e^	7.61±0.19^b^	6.71±0.09^c^	7.85±0.14^b^	11.82±0.31^a^

Note: values with distinct superscript letters within the same row were statistically significant (*p* < 0.05). All findings are shown as the mean ± standard deviation (*n* = 3).

**Table 4:** Biogenic amines contents of different fermented rice wines (mg/L).

	MJ1	MJ2	MJ3	MJ4	MJ5	MJ6	MJ7
Histamine	4.32±0.05^f^	3.04±0.07^g^	5.49±0.09^e^	6.12±0.06^d^	14.39±0.12^a^	9.26±0.21^b^	7.11±0.14^c^
Putrescine	27.19±0.39^d^	7.59±0.05^f^	47.23±1.02^a^	12.58±0.07^e^	39.15±1.20^c^	42.59±0.78^b^	46.39±0.86^a^
Cadaverine	1.32±0.04^f^	0.61±0.02^g^	4.53±0.06^a^	2.05±0.07^e^	4.17±0.13^b^	3.79±0.08^c^	2.76±0.06^d^
Tryptamine	0.42±0.02^g^	0.82±0.01^e^	0.54±0.01^f^	0.91±0.03^d^	2.35±0.05^b^	6.15±0.10^a^	1.49±0.04^c^
Tyramine	10.64±0.31^e^	6.73±0.03^f^	16.75±0.07^d^	25.52±0.11^c^	27.42±0.16^b^	57.38±0.74^a^	17.28±0.12^d^
Spermidine	0.73±0.01^e^	0.62±0.01^f^	1.29±0.02^b^	0.98±0.03^c^	2.62±0.02^a^	2.58±0.04^a^	0.91±0.02^d^
Spermine	2.38±0.07^a^	0.34±0.01^f^	0.82±0.02^e^	1.65±0.03^b^	1.27±0.03^c^	2.43±0.05^a^	0.94±0.04^d^
Phenethylamine	1.14±0.02^f^	0.72±0.02^g^	3.78±0.03^e^	5.34±0.04^d^	6.45±0.05^c^	8.36±0.06^a^	7.49±0.05^b^
Total	48.14±0.72^f^	20.47±0.13^g^	80.42±1.14^d^	55.15±0.08^e^	97.82±1.32^b^	132.54±1.66^a^	84.38±0.69^c^

Note: values with distinct superscript letters within the same row were statistically significant (*p* < 0.05).All findings are shown as the mean ± standard deviation (*n* = 3).

**Table 5:** Volatile compound composition and content in fermented rice wines (μg/L)

**Flavor components**	MJ1	MJ2	MJ3	MJ4	MJ5	MJ6	MJ7
**Alcohols**							
1-Propanol	22.03±1.33^e^	9.78±0.16^f^	38.10±1.15^c^	38.87±0.46^c^	35.65±0.48^d^	64.65±2.36^a^	45.03±2.07^b^
Isobutanol	654.39±11.01^a^	364.24±27.15^e^	403.17±31.20^d^	299.14±19.91^f^	524.70±19.60^b^	460.92±19.55^c^	557.97±9.51^b^
1-Butanol	11.40±0.27^c^	6.82±0.50^d^	7.68±0.41^d^	7.41±0.34^d^	25.60±1.69^a^	12.49±0.85^c^	14.07±1.10^b^
Isoamyl alcohol	4731.32±361.83^a^	2139.91±69.64^d^	2323.63±146.19^d^	2513.79±103.79^d^	4993.27±244.58^a^	4287.02±354.10^b^	3833.57±254.75^c^
Hexanol	ND	123.62±6.62^b^	119.61±11.34^b^	148.17±2.06^a^	ND	ND	ND
Heptanol	13.82±0.67^c^	20.98±1.63^b^	19.70±0.87^b^	21.94±0.55^b^	ND	14.75±1.02^c^	32.37±1.99^a^
2-Ethylhexanol	50.06±1.77^b^	10.84±0.51^d^	16.63±0.97^c^	14.45±0.81^c^	55.62±1.05^a^	55.41±3.01^a^	11.33±0.50^d^
2-Nonanol	4.58±0.15^c^	6.04±0.57^b^	8.87±0.45^a^	ND	ND	ND	9.10±0.68^a^
2,3-Butanediol	220.99±2.74^b^	ND	51.77±0.58^f^	145.71±3.66^c^	373.69±4.85^a^	98.86±9.64^e^	109.11±7.53^d^
Linalool	5.30±0.34^bc^	ND	15.77±0.89^b^	4.33±0.14^bc^	227.96±19.32^a^	17.14±1.61^b^	10.65±0.86^bc^
Octanol	61.02±4.11^b^	44.31±1.84^d^	52.78±3.26^c^	95.75±2.28^a^	49.96±1.85^c^	23.09±1.89^f^	33.00±2.04^e^
2-Octen-1-ol	4.52±0.26^a^	ND	ND	2.48±0.09^b^	ND	ND	ND
Nonanol	16.55±0.51^c^	15.08±1.14^d^	13.31±0.71^e^	24.93±0.45^a^	15.82±0.37^cd^	22.71±1.12^b^	16.17±0.21^cd^
Terpineol	7.51±0.30^c^	ND	ND	5.55±0.36^c^	350.53±6.04^a^	ND	16.76±0.84^b^
Methylthiopropanol	11.44±1.12^c^	ND	ND	8.26±0.46^d^	41.22±3.34^a^	14.45±1.04^b^	11.22±0.73^c^
Decanol	3.30±0.27^c^	14.94±0.50^b^	95.08±7.89^a^	14.61±0.39^b^	ND	12.41±0.98^b^	16.52±1.29^b^
Undecanol	ND	7.79±0.36^b^	64.24±4.10^a^	6.46±0.21^b^	ND	ND	ND
Citronellol	ND	ND	ND	ND	9.47±0.13^b^	43.68±2.22^a^	ND
Benzyl alcohol	16.68±1.54^b^	13.20±1.20^c^	19.47±0.59^a^	6.46±0.53^d^	12.40±0.50^c^	5.62±0.49^d^	19.68±1.21^a^
β-Phenylethanol	3592.63±150.36^d^	2153.98±146.21^f^	2495.68±217.11^f^	2629.66±63.29^e^	8456.95±307.84^a^	5758.81±233.54^b^	4354.49±166.35^c^
Lauryl alcohol	84.60±1.66^c^	185.90±12.12^b^	1147.81±104.76^a^	72.50±6.49^c^	1142.32±18.80^a^	35.87±2.12^c^	15.24±0.35^c^
Ionol	3.59±0.18^b^	ND	ND	ND	129.57±5.53^a^	ND	ND
Hexadecanol	ND	33.11±2.39^a^	18.11±1.54^b^	ND	7.16±0.16^c^	ND	ND
Nerolidol	ND	7.80±0.49^a^	5.86±0.43^b^	ND	ND	ND	ND
α-Cadinol	ND	ND	1.38±0.06^c^	5.39±0.34^b^	34.79±1.79^a^	ND	ND
Spathulenol	27.59±2.03^c^	34.43±3.04^b^	32.65±1.89^b^	54.66±4.56^a^	ND	ND	5.41±0.14^d^
α-Santalol	ND	1.18±0.06^c^	11.66±0.63^b^	13.33±0.53^a^	ND	ND	ND
α-Bisabolol	8.82±0.12	ND	ND	ND	ND	ND	ND
**Esters**							
Ethyl ascetate	204.95±3.77^cd^	46.77±2.03^e^	66.46±4.40^e^	117.82±0.78^de^	1619.27±148.33^a^	459.97±35.58^b^	252.82±6.73^c^
Ethyl isobutyrate	1.76±0.04	ND	ND	ND	ND	ND	ND
Isoamyl acetate	139.03±6.81^b^	55.48±4.94^c^	34.18±2.82^d^	62.67±5.28^c^	139.02±4.73^b^	160.54±8.65^b^	295.48±28.57^a^
Ethyl caproate	26.25±1.05^e^	48.45±3.65^d^	ND	160.92±7.36^b^	275.64±8.79^a^	135.17±9.26^c^	127.41±8.48^c^
Ethyl heptanoate	ND	ND	ND	4.44±0.43	ND	ND	ND
Ethyl pyruvate	ND	ND	ND	ND	18.40±1.08^a^	17.57±0.46^a^	ND
Ethyl lactate	216.04±0.77^c^	ND	ND	ND	750.25±11.94^a^	317.84±11.02^b^	142.29±1.07^d^
Ethyl caprylate	6.23±0.19^e^	3.51±0.33^e^	ND	82.02±5.52^d^	95.55±8.82^c^	477.35±1.42^a^	257.71±4.22^b^
Ethyl leucate	57.20±2.72^b^	15.15±1.11^c^	12.08±1.08^cd^	3.88±0.08^e^	197.74±6.66^a^	8.92±0.71^de^	6.21±0.15^e^
Heptyl formate	ND	ND	ND	ND	1.54±0.05^b^	12.78±0.80^a^	ND
Isobutyl lactate	ND	2.83±0.27^c^	3.21±0.23^c^	5.35±0.22^b^	3.08±0.19^c^	12.03±1.07^a^	5.07±0.14^b^
Amyl hexanoate	ND	ND	ND	13.85±0.79^a^	23.10±0.61^b^	ND	ND
Ethyl 3-hydroxybutyrate	5.84±0.30	ND	ND	ND	ND	ND	ND
Ethyl nonanoate	6.73±0.61^a^	ND	ND	ND	ND	ND	2.29±0.17^b^
γ-Butyrolactone	9.47±0.34^e^	28.48±2.46^c^	32.87±2.58^b^	37.58±3.67^a^	ND	13.88±0.69^d^	ND
Ethyl decanoate	22.18±1.88^c^	ND	ND	78.76±5.96^b^	82.62±6.07^b^	293.91±12.52^a^	5.49±0.25^d^
Diethyl succinate	757.02±32.27^b^	128.71±6.01^f^	228.75±0.79^de^	193.97±2.89^e^	1407.65±50.61^a^	426.16±19.55^c^	244.44±15.44^d^
Ethyl benzoate	ND	9.07±0.83^d^	ND	12.47±0.74^c^	26.01±2.60^a^	15.15±0.74^b^	1.42±0.09^e^
Ethyl glutarate	ND	ND	ND	1.56±0.14^b^	17.68±1.01^a^	ND	ND
Ethyl phenylacetate	22.01±1.23^c^	4.42±0.39^e^	5.94±0.23^e^	15.20±0.46^d^	168.37±3.16^a^	33.62±2.05^b^	24.41±1.39^c^
Phenethyl acetate	458.03±3.99^d^	176.13±10.96^f^	123.25±4.98^g^	326.94±11.11^e^	1066.68±28.51^a^	723.84±27.31^b^	628.90±20.51^c^
Ethyl laurate	ND	ND	ND	ND	ND	62.75±5.90	ND
Ethyl phenylpropionate	ND	0.94±0.04^b^	ND	ND	50.50±1.46^a^	ND	ND
Phenethyl isobutyrate	ND	ND	ND	ND	15.19±0.19^a^	3.99±0.39^b^	2.55±0.15^c^
Diethyl pimelate	6.02±0.52^b^	1.82±0.10^c^	0.96±0.04^d^	2.32±0.07^c^	20.62±1.11^a^	ND	ND
Ethyl myristate	134.05±5.77^b^	11.75±1.05^d^	8.44±0.72^de^	163.38±9.73^a^	ND	76.96±7.58^c^	3.91±0.35^de^
γ-Octanolactone	3.29±0.31^c^	ND	ND	ND	10.26±0.91^a^	ND	4.83±0.46^b^
Diethyl suberate	ND	ND	ND	5.73±0.51	ND	ND	ND
Ethyl pentadecanoate	ND	ND	ND	2.66±0.12	ND	ND	ND
γ-Decanolactone	9.37±0.46^b^	ND	ND	ND	154.94±3.60^a^	ND	ND
Diethyl azelate	ND	ND	ND	15.52±1.28^b^	28.85±1.24^a^	ND	2.78±0.16^c^
Ethyl palmitate	12.52±0.43^d^	18.34±1.22^d^	35.81±2.95^cd^	621.93±45.63^a^	53.99±5.40^c^	399.18±8.86^b^	15.98±0.50^d^
Ethyl palmitoleate	ND	ND	ND	8.67±0.72	ND	ND	ND
Hexyl acetate	ND	ND	1.91±0.05^b^	7.59±0.38^a^	ND	ND	ND
Methyl stearate	7.45±0.39^b^	4.95±0.29^bc^	2.58±0.24^cd^	ND	52.94±3.80^a^	ND	5.83±0.43^b^
Ethyl stearate	64.10±3.08^c^	6.41±0.41^e^	5.61±0.46^e^	12.87±0.21^d^	282.08±3.48^a^	113.64±6.35^b^	65.16±5.07^c^
Ethyl oleate	ND	3.41±0.10^cd^	4.92±0.37^cd^	167.70±8.75^a^	25.54±1.36^b^	171.75±9.23^a^	10.53±0.80^c^
Ethyl linoleate	ND	5.49±0.27^c^	3.75±0.29^c^	221.74±2.46^a^	ND	158.36±10.13^b^	5.74±0.45^c^
Ethyl linolenate	ND	ND	ND	7.64±0.61	ND	ND	ND
**Ketones**							
2-Octanone	92.31±6.49^b^	86.67±4.76^b^	89.77±6.87^b^	108.52±4.25^a^	75.70±6.74^c^	69.36±3.90^c^	97.54±6.53^b^
Acetoin	ND	ND	ND	ND	117.80±10.59^b^	583.47±16.37^a^	ND
2-Nonanone	ND	9.44±0.74	ND	ND	ND	ND	ND
3-Nonen-2-one	ND	5.40±0.50^a^	5.09±0.35^a^	2.97±0.11^b^	ND	ND	ND
Acetophenone	ND	ND	ND	5.94±0.21^a^	ND	2.11±0.20^c^	4.90±0.24^b^
2,3-Butanedione	14.85±0.17^a^	3.95±0.26^c^	ND	9.82±0.53^b^	ND	ND	ND
Phenylacetone	ND	ND	ND	ND	15.57±0.55^a^	5.19±0.21^b^	ND
β-Ionone	73.62±3.68^b^	ND	ND	ND	973.66±19.92^a^	ND	ND
Geranyl acetone	31.94±0.16^b^	9.96±0.90^e^	ND	14.25±0.84^d^	50.28±3.71^a^	20.16±1.94^c^	32.93±3.07^b^
α-Ionone	69.11±5.25^b^	ND	ND	ND	1542.16±45.70^a^	ND	ND
**Aldehydes**							
Nonanal	13.60±0.42^d^	21.07±1.49^c^	15.86±1.26^d^	32.74±1.79^a^	33.75±2.49^a^	33.17±1.55^a^	26.63±0.87^b^
2-Octenal	ND	13.41±0.74^a^	2.77±0.25^b^	2.97±0.11^b^	ND	ND	ND
Furfural	5.67±0.10^c^	5.12±0.44^c^	4.52±0.03^cd^	5.97±0.49^c^	182.96±6.74^a^	31.37±1.68^b^	ND
Benzaldehyde	ND	74.99±7.41^b^	55.29±3.16^c^	68.94±5.87^b^	ND	268.03±12.66^a^	ND
2,4-Nonadienal	ND	10.18±0.50^a^	7.98±0.79^b^	5.81±0.21^c^	ND	ND	ND
2,4-Decadienal	ND	6.17±0.50^b^	2.21±0.16^c^	6.90±0.68^a^	ND	ND	ND
Veratraldehyde	ND	736.26±63.06	ND	ND	ND	ND	ND
Cuminaldehyde	7.29±0.37^bc^	6.47±0.54^c^	5.00±0.14^d^	8.61±0.15^a^	3.51±0.13^e^	8.06±0.79^ab^	8.40±0.75^a^
5-Hydroxymethylfurfural	7.92±0.12^b^	ND	ND	ND	51.79±5.08^a^	ND	ND
**Acids**							
Acetic acid	64.22±2.12^cd^	48.52±1.32^de^	32.76±2.88^e^	53.82±3.49^cd^	317.33±22.91^a^	156.96±11.02^b^	68.24±4.12^c^
Isobutyric acid	26.87±1.66^c^	22.00±2.00^cd^	24.66±0.94^cd^	12.82±0.86^e^	78.65±7.09^a^	20.31±1.40^d^	33.09±1.59^b^
n-Butyric acid	4.59±0.40^c^	3.90±0.34^d^	3.55±0.22^e^	5.12±0.40^c^	11.75±1.00^b^	13.57±0.58^a^	4.76±0.39^c^
2,4-Dimethylvaleric acid	36.74±1.26^a^	8.19±0.07^d^	7.27±0.26^d^	25.83±1.54^b^	ND	ND	23.28±1.90^c^
Valeric acid	ND	ND	ND	ND	ND	135.79±3.03	ND
Caproic acid	98.27±5.26^d^	215.51±6.27^b^	ND	188.71±11.13^c^	471.57±3.34^a^	85.96±4.75^d^	174.59±17.01^c^
Heptanoic acid	ND	1.53±0.13^d^	3.13±0.14^b^	2.50±0.22^c^	ND	ND	5.58±0.30^a^
Caprylic acid	349.19±20.32^c^	101.65±0.95^d^	100.55±4.41^d^	94.35±3.17^d^	732.06±14.71^b^	913.19±24.40^a^	897.52±37.57^a^
Nonanoic acid	31.35±0.74^b^	23.04±2.26^c^	16.54±1.33^d^	22.87±2.27^c^	58.57±2.53^a^	23.50±1.92^c^	31.96±1.15^b^
Decanoic acid	73.54±0.91^d^	23.74±2.17^e^	31.50±1.96^e^	33.73±0.98^e^	741.08±31.98^a^	522.05±32.56^b^	190.15±8.25^c^
Benzoic acid	12.72±1.10^b^	8.47±0.54^c^	5.56±0.29^d^	ND	21.58±1.62^a^	ND	6.41±0.42^d^
Lauric acid	8.00±0.33	ND	ND	ND	ND	ND	ND
Tetradecanoic acid	ND	3.07±0.15^c^	ND	22.47±1.78^b^	26.98±2.55^a^	ND	ND
**Phenols**							
4-Ethylphenol	16.28±0.80^d^	16.03±1.49^d^	32.91±1.73^c^	181.85±6.80^a^	109.23±2.98^b^	ND	ND
**Olefins**							
3-Decene	11.08±0.94	ND	ND	ND	ND	ND	ND
Styrene	16.39±0.58^b^	5.82±0.56^c^	ND	26.97±1.39^a^	ND	ND	ND
**Alkanes**							
4-Decane	ND	ND	ND	23.57±1.74	ND	ND	ND
Undecane	ND	ND	ND	5.57±0.31^b^	17.28±0.77^a^	2.81±0.16^c^	0.76±0.04^d^
Hexadecane	ND	ND	7.32±0.40^a^	1.93±0.04^b^	ND	ND	1.69±0.07^b^

Note: values with distinct lowercase characters within the same row were statistically significant (*p* < 0.05). All findings are shown as the mean ± standard deviation (*n* = 3). ND, not detected.

In Section 2.6, the expression was incorrectly written as “Fluorescence detection (FLD) was performed at excitation and emission wavelengths of 254 nm”, the correct expression should be “Ultraviolet detection was performed at a wavelength of 254 nm”.

In Section 3.1, the value of ANN in MJ5 was incorrectly written as “27.03”, the correct value should be “27.13”.

In Section 3.2, the units for four organic acids were incorrectly written as “mg/L”, the correct unit should be “mg/100mL”.

In Section 3.4, the expression was incorrectly written as “118 aroma compounds were identified in the seven rice wine samples: 29 alcohols, 47 esters, 19 ketones and aldehydes, 13 acids, 4 phenols, 2 alkenes, and 4 alkanes”, the correct expression should be “105 aroma compounds were identifed in the seven rice wine samples: 28 alcohols, 39 esters, 19 ketones and aldehydes, 13 acids, 1 phenol, 2 olefins, and 3 alkanes”.

We regret that a very small portion of the data and significance analysis was incorrect due to copy-paste errors. The calculated data and significance analysis have been corrected in table 1-5. The corrected part are shown in red (attached below).

None of the corrections listed above affects the data, results, interpretation, or conclusions of the article.

The authors understand the significance of maintaining the integrity and accuracy of scientific publications, and we sincerely apologize for this oversight and any inconvenience caused to readers and the journal's editorial staff.

See Tables 1–5 below.

